# Endothelial Gata5 transcription factor regulates blood pressure

**DOI:** 10.1038/ncomms9835

**Published:** 2015-11-30

**Authors:** Smail Messaoudi, Ying He, Alex Gutsol, Andrew Wight, Richard L. Hébert, Ragnar O. Vilmundarson, Andrew P. Makrigiannis, John Chalmers, Pavel Hamet, Johanne Tremblay, Ruth McPherson, Alexandre F. R. Stewart, Rhian M. Touyz, Mona Nemer

**Affiliations:** 1 Faculty of Medicine, Department of Biochemistry, Microbiology and Immunology, University of Ottawa, 451 Smyth Road, Ottawa, Ontario, Canada K1H8M5; 2 Kidney Research Center, University of Ottawa, 451 Smyth Road, Ottawa, Ontario, Canada K1H8M5; 3 University of Ottawa Heart Institute, University of Ottawa, 451 Smyth Road, Ottawa, Ontario, Canada K1H8M5; 4 The George Institute for Global Health, The University of Sydney, The Royal Prince Alfred Hospital, 83-117 Missenden Road, Camperdown, New South Wales 2050, Australia; 5 Centre de Recherche du Centre Hospitalier de l'Université de Montréal, 900 rue St-Denis, Montréal, Québec, Canada H2X 0A9; 6 Institute of Cardiovascular and Medical Sciences, BHF Glasgow Cardiovascular Research Centre, University of Glasgow, 126 University Place, Glasgow G128TA, UK

## Abstract

Despite its high prevalence and economic burden, the aetiology of human hypertension remains incompletely understood. Here we identify the transcription factor GATA5, as a new regulator of blood pressure (BP). GATA5 is expressed in microvascular endothelial cells and its genetic inactivation in mice (*Gata5*-null) leads to vascular endothelial dysfunction and hypertension. Endothelial-specific inactivation of *Gata5* mimics the hypertensive phenotype of the *Gata5*-null mice, suggestive of an important role for GATA5 in endothelial homeostasis. Transcriptomic analysis of human microvascular endothelial cells with GATA5 knockdown reveals that GATA5 affects several genes and pathways critical for proper endothelial function, such as PKA and nitric oxide pathways. Consistent with a role in human hypertension, we report genetic association of variants at the *GATA5* locus with hypertension traits in two large independent cohorts. Our results unveil an unsuspected link between GATA5 and a prominent human condition, and provide a new animal model for hypertension.

## Abstract (web summary)

Unravelling the molecular basis of hypertension remains a major challenge. Here, the authors identify the transcription factor GATA5 as a novel regulator of blood pressure and potential genetic determinant of human hypertension and describe a unique mouse model for research of salt-sensitive hypertension.

## Graphical abstract

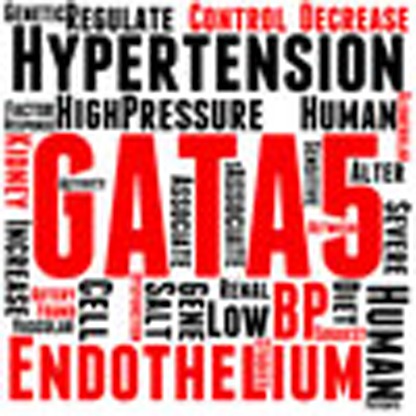

Hypertension is the most frequent cardiovascular risk factor with a prevalence of 25–30% worldwide^1^. High blood pressure (BP) is a well-established risk factor for cardiovascular morbidity and mortality associated with coronary artery disease (CAD), heart failure, stroke, as well as progression of chronic kidney disease^2^. An estimated 7 million deaths and 64 million disability-adjusted life years annually are related to poorly controlled hypertension^3^. BP is a complex, genetically determined trait, with estimates of heritability ranging from 31 to 68% (ref. 4). In the last 2 decades, large-scale genomic approaches have been used to identify genes/variants responsible for BP regulation. In particular, genome-wide association studies (GWAS) identified several loci in or near genes, many of which were previously not suspected of controlling BP^5^. However, despite significant progress, the collective effect of all BP loci identified through GWAS explains only a small fraction (2%) of BP heritability^5^. The genetic basis and pathophysiology of hypertension remain therefore obscure.

Candidate gene approaches and genetic manipulation of animal models have greatly contributed to the identification of the genetic basis of several complex human traits. Among others, these approaches have been particularly successful at uncovering genes linked to human congenital heart disease^6^. This includes *GATA5* in which several mutations have been found in patients with bicuspid aortic valves^7^,^8^ after this abnormality was reported in *Gata5*-null mice^9^. GATA5 is a member of the GATA family of transcription factors that regulate various aspects of cardiovascular cell expansion and differentiation^10^. Within the cardiovascular system, GATA5 is broadly but transiently expressed in endocardial cells and in endocardial cushion cells of the atrio-ventricular canal and outflow tract during embryogenesis^11^,^12^. *In vitro*, GATA5 is necessary for differentiation of cardiogenic precursors into endothelial/endocardial cells^13^.

We report that GATA5 is present in microvascular endothelial cells and that its absence in mice leads to increased BP, endothelial dysfunction and age-dependent end-organ damage, which are all features of human hypertension. Mechanistically, loss of GATA5 disrupts several pathways essential for proper endothelial signalling and homeostasis. Consistent with a possible role in human hypertension, we find genetic association of variants within the *GATA5* locus with prescription of anti-hypertensive medication in two large independent cohorts. Thus, *GATA5* may be a susceptibility locus for hypertension and possibly other endothelial-dependent human disorders.

## Results

### Loss of GATA5 in mice leads to hypertension

Inactivation of *Gata5* in mice leads to a partially penetrant bicuspid aortic valve^9^. Full penetrance of concentric cardiac hypertrophy^9^ and increased *Nppa* (encoding atrial natriuretic peptide) expression suggest the existence in these mice of additional cardiovascular alterations (Supplementary Fig. 1). Cardiac hypertrophy is often an adaptive response to pressure overload^14^ and is thus a frequent condition in patients with hypertension^15^. We therefore measured BP in *Gata5*-null mice.

**Figure 1:**
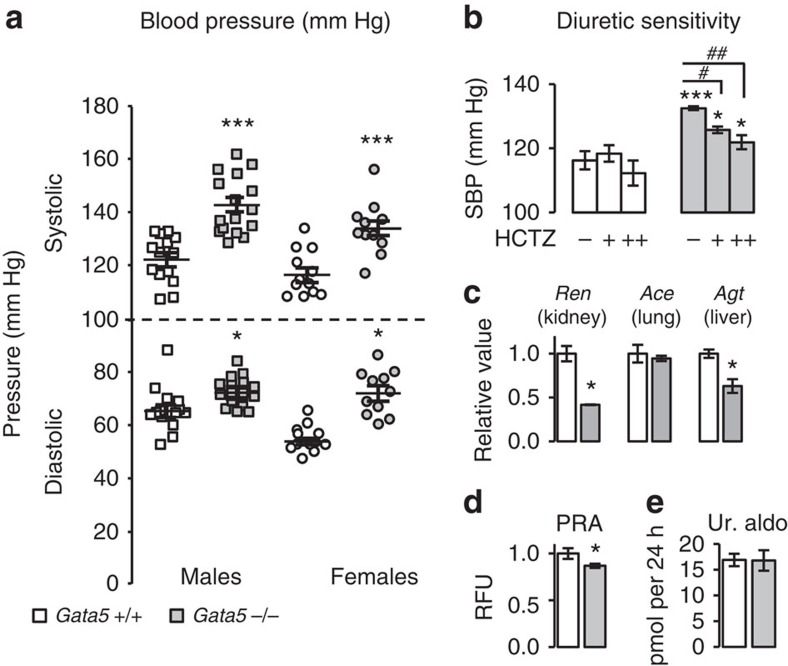
*Gata5*-null mice have low-renin hypertension. (**a**) Increased systolic and diastolic BP in 90-day-old *Gata5*-null mice. Both genders are hypertensive (males *n*=14–16 per group; females *n*=11–12 per group). The results are reported as mean±s.e.m. *P<0.05; ***P<0.005 (two-factor ANOVA). (**b**) Hypertension in *Gata5*-null mice is sensitive to diuretic: hydrochlorothiazide (HCTZ) for 5 days lowered, but did not normalize BP in *Gata5*-null mice at both low (+, 2.8 mg per day) and high dose (++, 8 mg per day) (*n*=4 per group). The results are reported as mean±s.e.m. *P<0.05 versus *Gata5*+/+ mice; ***P<0.01 versus *Gata5*+/+ mice; ^#^*P*<0.05 versus untreated mice, ^##^*P*<0.01 versus untreated mice (repeated measures two-factor ANOVA test followed by Bonferonni correction for multiple comparisons). (**c**) Low-renin hypertension in *Gata5*-null mice: the expression of *Ace* (angiotensin-converting enzyme) gene in the lung was unchanged, while expression of *Ren* (renin) in the kidney and *Agt* (angiotensinogen) gene in the liver was decreased. The expression of these genes was measured by qPCR (*n*=3–4 per group). The results are reported as mean±s.e.m. *P<0.05 versus *Gata5*+/+ mice (Mann–Whitney test). (**d**) Plasma renin activity (PRA) was decreased in *Gata5*-null mice versus their control littermates (*n*=8–10 per group). The results are reported as mean±s.e.m. *P<0.05 versus *Gata5*+/+ mice (*t*-test). (**e**) There are no changes in aldosterone urinary concentration as measured by the enzyme immunoassay. (*n*=6 per group). The results are reported as mean±s.e.m. (*t*-test).

Both systolic and diastolic BP were significantly increased in young male and female *Gata5*-null mice (90 days old) when compared with their control littermates (Fig. 1a). Hydrochlorothiazide (HCTZ), a widely used diuretic in the treatment of hypertension^16^ reduced, but did not normalize BP at either dose used (2.7 and 8 mg per day, respectively) (Fig. 1b). This effect is similar to that observed in human hypertension^17^. Of note, the expression and localization of the HCTZ target (the sodium chloride co-transporter) was similar between *Gata5*-null mice and their controls (Supplementary Fig. 2).

The renin–angiotensin–aldosterone system is the main hormonal regulator of BP and its activation leads to hypertension. Expression and/or activity of several renin–angiotensin–aldosterone system components were measured and were either unchanged or decreased in *Gata5*-null mice. The expression of the angiotensin-converting enzyme (*Ace*) gene in the lung was unchanged, whereas the hepatic expression of the Angiotensinogen (*Agt*) gene was decreased, as was the renal expression of the Renin (*Ren*) gene (Fig. 1c). Plasma renin activity was also slightly but significantly decreased in *Gata5*-null mice (Fig. 1d). No changes were observed in urinary aldosterone concentrations (Fig. 1e).

**Figure 2:**
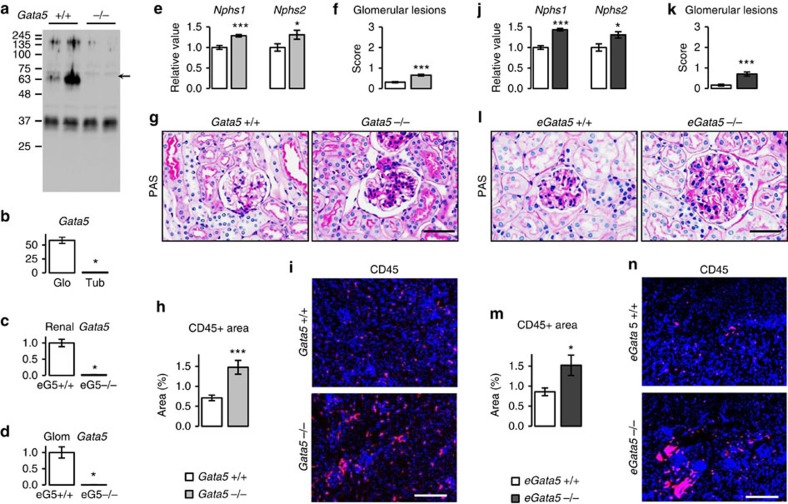
Loss of Gata5 from renal endothelial cells leads to renal alterations. (**a**) GATA5 is expressed in the kidney as assessed by western blot performed on total kidney extracts. (**b**) *Gata5* is essentially expressed in the glomeruli as assessed by qPCR on isolated glomeruli (Glo) and microdissected tubules (Tub) from Wt mice kidneys. (*n*=3–5 per group). The results are reported as mean±s.e.m. *P<0.05 versus *Gata5*+/+ mice (Mann–Whitney test). (**c**,**d**) Specific deletion of *Gata5* in endothelial cells (e*Gata5-null* mice) virtually abolished renal and glomerular expression of *Gata5* (*n*=3 per group). The results are reported as mean±s.e.m. *P<0.05 versus *eGata5*+/+ mice (Mann–Whitney test). (**e**) The expression of the glomerular genes *Nphs1* (nephrin) and *Nphs2* (podocin) as measured by qPCR (*n*=6 per group) was increased in *Gata5*-null mice. The results are reported as mean±s.e.m. *P<0.05 versus *Gata5*+/+ mice; ***P<0.005 versus *Gata5*+/+ mice (*t*-test for nephrin; Mann–Whitney test for podocin). (**f**–**i**) Absence of *Gata5* induces glomerular lesions (sections are stained with periodic acid Schiff; scale bar, 30 μm; *n*=5 per group) and renal inflammation as assessed by leucocytes CD45 immunostaining (scale bar, 200 μm; *n*=4 per group). The results are reported as mean±s.e.m. ***P<0.005 versus *Gata5*+/+ mice (Mann–Whitney test). (**j**–**n**) Deletion of *Gata5* from endothelial cells reproduces the renal phenotype of global *Gata5* deletion: both *Nephs1* and *Nphs2* transcript levels were increased (*n*=4–6 per group) as well as glomerular lesion score (scale bar, 30 μm; *n*=4–6 per group) and renal leucocytes infiltration (scale bar, 200 μm; *n*=4–5 per group) in e*Gata5*-null mice in comparison with their controls. The results are reported as mean±s.e.m. *P<0.05 versus e*Gata5*+/+ mice; ***P<0.005 versus e*Gata5*+/+ mice (Mann–Whitney test).

Since impaired kidney handling of salt and water balance is a key factor in the pathophysiology of hypertension^18^, we analysed GATA5 role therein. GATA5 is expressed in the kidney (Fig. 2a) where it might affect electrolyte homeostasis. To test this possibility, *Gata5*-null mice and their controls were maintained in metabolic cages and urine and blood samples were collected. At steady state, there were no differences in food and water intake between the two groups. Twenty-four hour urinary excretion of Na^+^, K^+^ and Cl^−^, as well as plasma electrolyte concentrations were also unchanged. Similarly, no differences in urinary electrolyte secretion between the *Gata5*-null mice and their controls were detected in response to 24-h acute salt loading (Supplementary Table 1). At steady state, the renal expression of co-transporters involved in sodium reabsorption was either unchanged or decreased in *Gata5*-null mice, suggesting pressure natriuresis compensatory mechanisms (Supplementary Figs 3a,b and 4). There were no changes in expression of components of the anti-natriuretic intra-renal angiotensin system or of the pro-natriuretic intra-renal dopaminergic system (Supplementary Fig. 3c,d). Loss of GATA*5* has therefore minimal effect on renal electrolyte homeostasis.

### *Gata5* is predominantly expressed in renal endothelial cells

To characterize the role of GATA5 within the kidney, we set out to determine its expression sites. Unfortunately, none of the antibodies we tested were suitable for immunohistochemistry, so we resorted to quantitative PCR (qPCR) analysis on isolated glomeruli and microdissected tubules (Supplementary Fig. 5). As shown in Fig. 2b, *Gata5* transcripts were enriched in the glomerulus (60 × versus nephron, *P*<0.05, Mann–Whitney test). Renal *Gata5* expression was reduced by 98% (*P*<0.001, Mann–Whitney test) and became virtually undetectable in kidneys and glomeruli of mice with endothelial-specific deletion of *Gata5* (e*Gata5*-null mice) (Fig. 2c,d), indicating that *Gata5* expression in the kidney is mostly endothelial. Consistent with this, the messenger RNA (mRNA) level of key glomerular markers such as nephrin (+29% versus controls, *P*<0.05, *t*-test) and podocin (+31% versus controls, *P*<0.05, Mann–Whitney test) was altered in *Gata5*-null mice (Fig. 2e) and glomerular cellularity, was significantly increased in these mice (Fig. 2f,g). Leucocytes infiltration was also increased twofold in renal cortex of *Gata5*-null mice as assessed by CD45 immunostaining, and they were found all around the tubules and the glomeruli (Fig. 2h,i). Flow cytometry analysis of the renal immune populations suggested that infiltrated CD45^+^ cells were macrophages, and that was confirmed by F4/80 kidney immunostaining (Supplementary Fig. 6a–c). No increase in CD45^+^ cells content was detected in other tissues (Supplementary Fig. 7), and the bone marrow and lymphoid organs (thymus and spleen) immune subsets' composition was similar between the *Gata5*-null mice and their controls (Supplementary Fig. 8a,c,e). The same renal and immune phenotype was observed in e*Gata5*-null mice (Fig. 2h–n; Supplementary Fig. 8b,d,f). Together, these data suggest that lack of GATA5 from endothelial cells promotes glomerular lesions and renal inflammation likely reflecting altered renal endothelial cross-talk with other cell types, which can contribute to hypertension development^19^.

### Endothelial *Gata5* is responsible for increased BP

**Figure 3:**
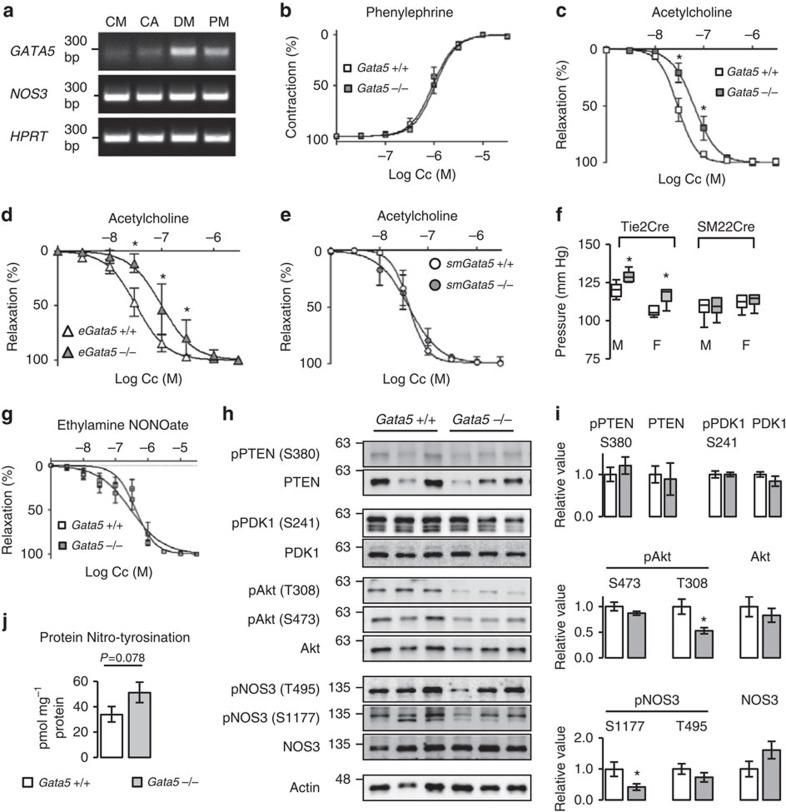
Endothelial *Gata5* is responsible for increased blood pressure. (**a**) *GATA5* is expressed in human cardiac microvascular (CM), coronary artery (CA), dermal microvascular (DM) and pulmonary microvascular (PM) endothelial cells. (**b**,**c**) The vasoconstrictor response of *Gata5*-null mice mesenteric arteries to norepinephrine is unaltered (*n*=7 per group), while the vasodilatory response to acetylcholine is decreased (*n*=10–11 per group). The results are reported as mean±s.e.m. *P<0.05 versus controls (comparison of best-fit values—effector concentration for half-maximum response (EC_50_) and Hill slope—using an F-test). (**d**–**f**) Deletion of *Gata5* in endothelial (e*Gata5*-null mice; *n*=4–5 per group) but not smooth muscle cells (sm*Gata5*-null mice; *n*=5 per group) decreases mesenteric arteries sensitivity to acetylcholine and increases BP (e*Gata5*-null mice *n*=6–10 per group; sm*Gata5*-null mice *n*=6–9 per group). The results are reported as mean±s.e.m. *P<0.05 versus controls (two-factor ANOVA). (**g**) The vasodilatory response of *Gata5*-null mice to diethylamine NONOate, an NO donor, is unaltered (*n*=7 per group). The results are reported as mean±s.e.m. (comparison of best-fit values—EC_50_ and Hill slope—using an F-test). (**h**,**i**) NOS3 and Akt phosphorylation are decreased in *Gata5*-null mice mesenteric arteries. PTEN and PDK1 phosphorylation and expression are unaltered (*n*=5–7 per group). Phosphorylated proteins are normalized to total proteins. Total proteins are normalized to actin. The results are reported as mean±s.e.m. *P<0.05 versus *Gata5*+/+ mice (*t*-test). (**j**) Quantification of protein nitrotyrosination in mesenteric arteries of *Gata5*-null mice and their controls as measured by ELISA. 3-Nitrotyrosine content is expressed as picomole of nitrotyrosine per milligram of protein (*n*=5–7 per group). The results are reported as mean±s.e.m. (*t*-test).

Presence of *Gata5* in glomerular endothelial cells raised the possibility that it may be expressed in other endothelial vascular beds. Indeed, *GATA5* was expressed in all the human vascular endothelial primary cell cultures tested with the highest level in peripheral vascular cells such as dermal microvascular endothelial cells (Fig. 3a; Supplementary Fig. 9). We therefore tested whether GATA5 was involved in vascular function. The vasoconstrictor response of mesenteric arteries to phenylephrine was similar in control and *Gata5*-null mice (Fig. 3b). In contrast, there was significant alteration in the vasodilatory response to acetylcholine with a twofold increase in the effector concentration for half-maximum response in *Gata5*-null mesenteric arteries, indicative of endothelial dysfunction (Fig. 3c). To confirm the role of endothelial GATA5 in vascular reactivity and BP regulation, we analysed mice with specific endothelial (e*Gata5*-null mice) or smooth-muscle (sm*Gata5*-null mice) *Gata5* deletion. The vasodilatory response to acetylcholine was decreased in mesenteric arteries from e*Gata5*-null, but not from sm*Gata5*-null mice (Fig. 3d,e). Similarly, BP was significantly elevated in mice lacking *Gata5* in endothelial but not in smooth muscle cells (Fig. 3f). These results indicate that the hypertensive phenotype of *Gata5*-null mice is mostly of endothelial origin.

The vasodilatory response of mesenteric arteries to ethylamine NONOate, an NO donor was tested. Unlike what was observed with acetylcholine, control and *Gata5*-null mice had a similar response, suggesting that *Gata5*-null mice have altered NO production or bioavailability not NO handling (Fig. 3g). The activity of the endothelial nitric oxide synthase (NOS3) is regulated by phosphorylation at multiple sites, and depends on diverse kinases and phosphatases such as Akt and PTEN^20^. In the mesenteric arteries of *Gata5*-null mice, the phosphorylation of NOS3 on Threonine 495 (inhibitory) was unchanged, whereas it was decreased by 58% on the critical activation site serine 1177, which would lead to decreased endothelial NO production (Fig. 3h,i; Supplementary Fig. 9). Phosphorylation of Akt was decreased by 50% on threonine 308, suggesting that decrease in NOS3 phosphorylation in *Gata5*-null mice mesenteric arteries could rely on deregulated Akt signalling. Classically, Akt is activated by PDK1, whereas PTEN is a major negative regulator of the Akt pathway^21^. No changes in PDK1 and PTEN expression and phosphorylation were observed, suggesting that loss of GATA5 affects Akt activation independently of PDK1 and PTEN. Furthermore, we also found a strong trend to increased protein nitrotyrosination (a hallmark of oxidative stress) in *Gata5*-null mice mesenteric arteries (*P*=0.076, *t*-test) (Fig. 3j), reflecting a possible imbalance in oxidative stress that may contribute to decreased NO bioavailability^22^.

**Figure 4:**
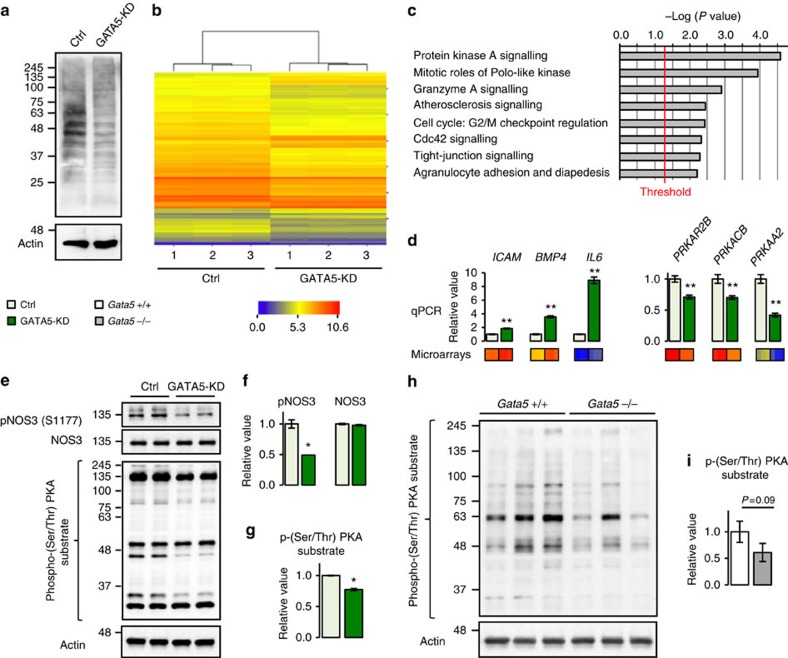
GATA5 regulates several pathways in endothelial cells. (**a**) GATA5 expression is significantly decreased in human dermal microvascular endothelial cells infected with a lentiviral vector containing an anti-*GATA5* shRNA (HDMEC-GATA5-KD). Control cells were infected with a vector containing a control shRNA (targets no known mammalian gene) (HDMEC-pLKO-Ctrl, referred here as Ctrl). (**b**) Heatmap representation of the differentially regulated genes between HDMEC-GATA5-KD cells and their controls as identified by transcriptomic analysis. Colour is function of Log2 RMA (Affymetrix microarray, *n*=3 per group). (**c**) Functional analysis of the differentially regulated genes between HDMEC-GATA5-KD cells and their controls. Protein kinase A pathway is the most significantly enriched pathway. Fisher's exact test *P* value. (**d**) Validation by qPCR (upper panel) of genes predicted by microarray (lower panel) to be up- and downregulated in HDMEC-GATA5-KD endothelial cells. (*n*=5 wells per condition). Downregulated genes: *PRKACB* codes for the PKA catalytic subunit β, *PRKAR2B* for the PKA regulator subunit 2β and *PRKAA2* for the AMPK catalytic subunit α2. Upregulated genes: *ICAM1* codes for the intercellular adhesion molecule 1, *BMP4* for the bone morphogenetic protein 4 and *IL6* for the interleukin 6. The results are reported as mean±s.e.m. **P<0.01 versus Ctrl (*t*-test). (**e**) Western blot representation of phospho-NOS3, NOS3 (Ser1177) and phospho-(Ser/Thr) PKA substrate motif in HDMEC-GATA5-KD cells and their controls. (**f**) Phosphorylation of NOS3 on Ser1177 is decreased in HDMEC-GATA5-KD cells (performed twice, 2–3 wells per condition). Phospho-NOS3 is normalized to total NOS3. NOS3 is normalized to actin. The results are reported as mean±s.e.m. *P<0.05 versus Ctrl (Mann–Whitney test). (**g**) Phosphorylation of (Ser/Thr) PKA substrate motif is decreased in HDMEC-GATA5-KD cells (performed twice, 2–3 wells per condition). Phospho-(Ser/Thr) PKA substrate motif (between 25 and 250 kDa) is normalized to actin. The results are reported as mean±s.e.m. *P<0.05 versus Ctrl (Mann–Whitney test). (**h**) Western blot representation of phospho-(Ser/Thr) PKA substrate motif in mesenteric arteries of *Gata5*-null mice and their controls. (**i**) In mesenteric arteries of *Gata5*-null mice, there is a trend to decrease in the (Ser/Thr) PKA substrate motif phosphorylation (*n*=4–5 per group). Phospho-(Ser/Thr) PKA substrate motif (between 25 and 250 kDa) is normalized to actin. The results are reported as mean±s.e.m. (Mann–Whitney test).

To further elucidate the role of GATA5 in endothelial cells, we generated a stable GATA5 knockdown cell line (Human Dermal Microvascular Endothelial Cell (HDMEC)-GATA5-KD) by infecting HDMECs with a lentiviral vector containing an anti-*GATA5* short hairpin RNA (shRNA) (Fig. 4a), followed by a transcriptomic analysis. HDMEC infected with a lentivirus containing a control shRNA (targets no known mammalian gene) served as control (HDMEC-pLKO-Ctrl). Transcriptomic analysis identified 649 genes (192 increased and 457 decreased, fold change ≥1.5 and *P*≤0.05, Mann–Whitney test with *P* value adjusted—Benjamini and Hochberg—for multiple comparisons) differentially regulated between HDMEC-GATA5-KD cells and their controls (Fig. 4b; Supplementary Table 2). Functional annotation of these genes using IPA Ingenuity showed enrichment of several pathways known to play important roles in endothelial homeostasis (Fig. 4c). The protein kinase A (PKA) pathway, an important regulator of NOS3 activation and endothelial function, was the most significantly enriched (*P*=2.9E−05, Fisher's exact test). Quantification by qPCR of several genes of the PKA pathway was consistent with microarray data (*PRKACB* coding for the PKA catalytic subunit β, *PRKAR2B* coding for the PKA regulator subunit 2β and *PRKAA2* coding for the AMPK catalytic subunit α2) (Fig. 4d). In line with the microarray data and the *in vivo* findings, HDMEC-GATA5-KD cells exhibited a significant decrease on NOS3 serine 1177 phosphorylation (Fig. 4e,f), and assessment of PKA activity by western blot using an antibody raised against phosphorylated serine/threonine PKA substrate motifs showed a decrease in the phosphorylation of many PKA targets therein (Fig. 4e–g). The mesenteric arteries of *Gata5*-null mice also revealed a decreased PKA activation trend (*P*=0.09, Mann–Whitney test) (Fig. 4h,i). Other important pathways for endothelial homeostasis that were disrupted included inflammatory pathways (for example, atherosclerosis, agranulocyte adhesion and diapedesis) represented by the upregulation of genes such as *ICAM1*, *BMP4* and *IL6* (Fig. 4d). Altogether, these results indicate that GATA5 regulates multiple pathways essential for endothelial homeostasis that can directly contribute to the onset and maintenance of hypertension.

**Figure 5:**
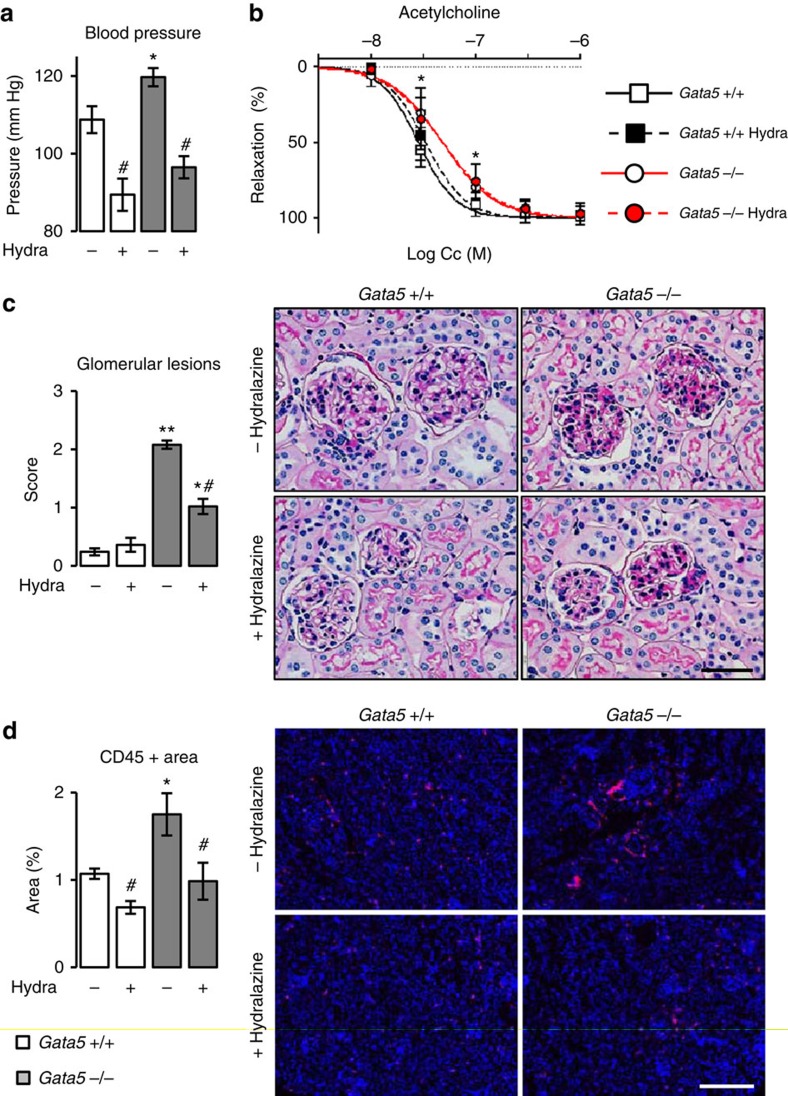
Effects of BP normalization on *Gata5*-null mice renal and vascular alterations. (**a**) Administration of hydralazine (a smooth muscle cell relaxant) for 4 weeks decreased blood pressure similarly in both *Gata5*-null mice and their controls (*n*=5–7 per group). The results are reported as mean±s.e.m. *P<0.05 versus controls; ^#^*P*<0.05 versus corresponding untreated mice (two-factor ANOVA followed by Bonferonni correction for multiple comparisons). (**b**) Hydralazine had no effect on *Gata5*-null mice endothelial dysfunction (*n*=5–7 per group). The results are reported as mean±s.e.m. *P<0.05 versus controls (comparison of best-fit values—EC_50_ and Hill slope—using an F-test). (**c**) Hydralazine decreased partially glomerular injuries in *Gata5*-null mice (sections are stained with periodic acid Schiff; scale bar, 30 μm; *n*=5–7 per group). *P<0.05 versus controls; ^#^*P*<0.05 versus corresponding untreated mice (two-factor ANOVA followed by Bonferonni correction for multiple comparisons). (**d**) Hydralazine decreased completely renal CD45+ cells infiltration in *Gata5*-null mice and also their controls (scale bar, 200 μm; *n*=5–7 per group). *P<0.05 versus controls; ^#^*P*<0.05 versus corresponding untreated mice (two-factor ANOVA followed by Bonferonni correction for multiple comparisons).

Finally, to confirm that endothelial dysfunction was inherent to loss of GATA5, not secondary to hypertension, we treated *Gata5*-null mice and their controls with hydralazine, a widely used vascular smooth muscle cell relaxant. Hydralazine led to a significant drop in BP in both groups (*P*<0.001, two-factor analysis of variance (ANOVA) test) but did not normalize endothelial dysfunction and reduced only partially glomerular lesions (Fig. 5a–c). This suggests that both endothelial dysfunction and kidney glomerular injuries in *Gata5*-null mice are not secondary to BP raise and are likely primary contributors to deregulated BP. In contrast, hydralazine abrogated renal infiltration of CD45+ cells, indicating that renal inflammation is secondary to increased BP (Fig. 5d).

### Ageing and hypertension complications

**Figure 6:**
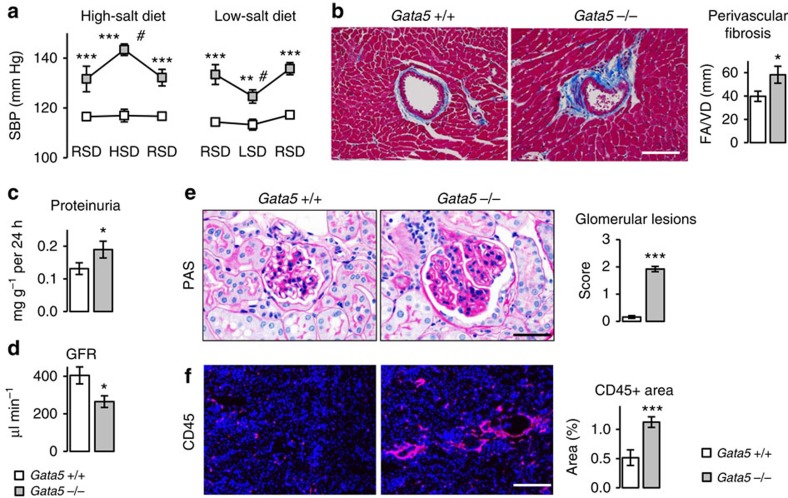
*Gata5*-null mice have features of human essential hypertension. (**a**) *Gata5*-null mice are salt sensitive: adult mice were fed either a high-salt diet (HSD, 8% NaCl) for 6 weeks or low-salt diet (LSD, 0.02% NaCl) for 10 days. HSD increased BP in *Gata5*-null mice, while LSD decreased it. A regular salt diet (RSD, 0.5% NaCl) for 4 weeks brought BP to its baseline (*n*=4 per group). The results are reported as mean±s.e.m. ***P<0.005 versus corresponding *Gata5*+/+ mice; ^#^*P*<0.05 versus *Gata5*+/+ mice fed with RSD (repeated measures two-factor ANOVA test followed by Bonferonni correction for multiple comparisons). (**b**) Increased perivascular fibrotic area to vessel diameter ratio (PFA/VD) in old *Gata5*-null mice. Sections are stained with Masson's Trichrome (scale bar, 100 μm; *n*=5–7 per group). The results are reported as mean±s.e.m. *P<0.05 versus *Gata5*+/+ mice (*t*-test). (**c**,**d**) Kidney dysfunction in 12-months-old *Gata5*-null mice is characterized by decreased glomerular filtration rate (GFR) and increased urinary protein excretion (*n*=5–6 per group). The results are reported as mean±s.e.m. *P<0.05 versus *Gata5*+/+ mice (*t*-test). (**e**) Periodic acid Schiff (PAS) staining showing significant alterations of glomerular structure in old *Gata5*-null mice (scale bar, 30 μm; *n*=5–7 per group). Quantification is on the right. The results are reported as mean±s.e.m. ***P<0.005 versus *Gata5*+/+ mice (*t*-test). (**f**) Significant increase in inflammatory cells infiltration in *Gata5*-null mice kidneys as assessed by CD45 (leucocytes) immunostaining (scale bar, 200 μm; *n*=5–7 per group). The results are reported as mean±s.e.m. ***P<0.005 versus *Gata5*+/+ mice (*t*-test).

Later stages of hypertension are generally associated with salt sensitivity and target-organ damage, in particular the heart and kidney^18^. We tested whether the evolution of hypertension in *Gata5*-null mice follows the natural history of human hypertension. A high-salt diet (8% NaCl) increased BP in 8-month-old *Gata5*-null mice, while a low-salt diet (0.025% NaCl) decreased it, without normalizing it (Fig. 6a). Regular salt diet for 4 weeks after the end of high- or low-salt diet treatment brought BP back to its initial level. Thus *Gata5*-null mice develop salt sensitivity with age. The older *Gata5-null* mice (on a regular salt diet) also developed cardiac perivascular fibrosis (Fig. 6b) and several features of hypertensive nephropathy with increased proteinuria and decreased glomerular filtration rate (Fig. 6c,d). Histology revealed segmental glomerulosclerosis with mesangial cell proliferation, glomerular basement membrane thickening and accumulation of extracellular matrix (Fig. 6e). Similarly, inflammatory (*Et1*, *Ccl2*, *Ccl5* and *Pai1*) and kidney injury (*Havcr1*) markers (Supplementary Fig. 10) as well as inflammatory cell infiltration were also increased in these older animals (Fig. 6f). The *Gata5*-null mice represent, therefore, a new model of systemic hypertension that reproduces several of the features found in hypertensive patients.

### *GATA5* variants and BP-related traits in humans

To test the clinical relevance of our findings, we asked whether genetic variants at the *GATA5* gene would reveal a nominal association with hypertension (defined as prescription of anti-hypertensive medication) in two large independent clinical data sets, the ADVANCE (Action in Diabetes and Vascular Disease: Peterax and Diamicron MR Controlled Evaluation) GWAS sub-study (*n*=2391) and the Ottawa Heart Genomics Study, OHGS (*n*=5835). The OHGS is a case–control study aimed at discovering genetic variants that modify the risk of CAD independent of diabetes (that is, diabetic patients were excluded from the study)^23^, whereas ADVANCE is a factorial, randomized controlled clinical trial assessing the effect of hypertension treatment and glucose-lowering drugs on diabetic complications^24^. Six variants within a region of 56 kb either side of *GATA5* were analysed (rs8113921, rs6061535, rs6142769, rs6587235, rs6587239 and rs6061245). Of the six variants studied, two are within *GATA5* and both were significantly associated with anti-hypertensive medication in both studies when the additive model was assumed: rs6061245 in intron 3 (ADVANCE: *P*=2E−03, beta=−0.188; OHGS *P*=0.031, beta −0.081, frequentist association test) and rs6587239 a synonymous polymorphism in exon 5 (ADVANCE: *P*=6E−04, beta=0.204; OHGS *P*=0.049, beta 0.075, frequentist association test) (Table 1). *In silico* analysis of *GATA5* exon 5 with Human Splicing Finder V3 (ref. 25) showed that rs6587239 was located within a region rich in splicing regulatory sites (SRSs) and that substitution of a guanine by an adenine breaks or creates a new SRS (Supplementary Fig. 11) and may therefore affect *GATA5* RNA splicing. Together these data suggest that *GATA5* might be a susceptibility gene for human hypertension.

## Discussion

The data presented identify GATA5 as a new regulator of BP. In mice, loss of GATA5 leads to a hypertensive phenotype that reproduces several of the features of human hypertension, including vascular dysfunction, salt sensitivity and target-organ damage. Association between *GATA5* sequence variants and use of hypertension medication in two large independent studies suggest that GATA5 might be relevant to human hypertension.

Our results demonstrate that GATA5 is expressed in adult microvascular endothelial cells, where it regulates numerous genes and pathways required for endothelial homeostasis and BP regulation. Clinical studies suggest that endothelial dysfunction plays a precursor role in the hypertensive pathophysiology. Endothelium-dependent vasodilation is impaired in the offspring of patients with hypertension^26^ and it has been shown that individuals with a positive family history of hypertension have an abnormal L-arginine uptake^27^. Impairment of endothelial function in patients with pre-hypertension^28^ further suggests that endothelial dysfunction may promote the development of hypertension. Animal models of hypertension also strongly support that concept. Experimental inhibition of NO, either pharmacologically by administration of L-NAME^29^ or genetically by inactivation of NOS3 (ref. 30) increases BP. In contrast, pharmacological enhancement of NOS activity restores endothelial function and decreases BP in spontaneously hypertensive rats^31^. In this respect, our finding that loss of GATA5 decreases vascular NO bioavailability is noteworthy. Transcriptomic analysis of endothelial cells suggest that this could be directly related to the decreased expression and activity of kinases such as PKA (PKA-cat β and PRKAR2) and AMPK (AMPKα2) that phosphorylate and activate NOS3 and its direct upstream activator Akt^32^. In endothelial cells, PKA directly phosphorylates NOS3 and induces production of nitric oxide in response to shear stress^33^, whereas β-adrenergic stimulation of NOS3 phosphorylation in bovine endothelial cells depends on PKA activation of Akt^34^. Similarly, AMPK activates NOS3 in response to various stimuli, and genetic inactivation of AMPKα2 attenuates atorvastatin-induced NOS3 phosphorylation *in vivo*^35^. In addition to their direct effect on NOS3, these kinases are central regulators of the stress response either through their effects on vascular wall integrity or on the production/buffering of reactive oxygen species (ROS). Thus, AMPKα2, which is significantly decreased in the absence of GATA5, is central to endothelial stress response including regulation of NADPH oxidase^36^,^37^. In fact, AMPKα2 acts as a physiological suppressor of ROS production and has also been shown to have anti-inflammatory effects on endothelial cells^38^. Similarly, PKA inhibits transcription of BMP4, which stimulates ROS production in endothelial cells and has been shown to have hypertensive and pro-inflammatory effects on the vasculature^39^,^40^. Of note, our *ex vivo* observations show that PKA activity is decreased, whereas BMP4 expression is upregulated in endothelial cells with GATA5 knockdown. In addition to alteration of several endothelial-signalling pathways, loss of GATA5 upregulated the expression of pro-inflammatory molecules (ICAM1, IL6) that would also be expected to contribute to endothelial dysfunction and to participate in maintaining the hypertensive phenotype and the inflammatory process that accompanies organ damage.

Noteworthy, the Tie2 promoter used to delete *Gata5* from endothelial cells is also active in the myeloid lineage^41^. However, our experimental observations suggest that GATA5 has no direct major effect on the immune system, and that macrophage infiltration in *Gata5-null* mice was secondary to increased BP and specific to the kidney, not the reflection of a systemic immune disorder. In fact, results from the Immunological Genome Project suggest that GATA5 is not expressed at an appreciable level in any immune population or stage of development^42^. Therefore, the phenotype of e*Gata*5-null mice most likely results from endothelial deletion of *Gata5* from endothelial cells and reflects GATA5 function in endothelial cells but not in other cells of the haematopoietic lineage.

Control of BP requires complex integration of regulatory mechanisms across multiple physiological systems (renal, cardiovascular, sympathetic and endocrine)^18^. By its effects on endothelial homeostasis, GATA5 may also affect BP through mechanisms that go beyond the vascular system. Pressure natriuresis is a key mechanism for long-term sodium and water balance control. Increasing BP is a mean of achieving these balances in the face of intra-renal disturbances such as reduction in glomerular filtration rate or increase in tubular reabsorption^43^. It is therefore possible that endothelial dysfunction also affects *Gata5*-null mice renal arteries and blood flow, leading to decreased renal perfusion as observed in pharmacological and genetic models of NOS inhibition^44^. This could shift the pressure natriuresis to higher BP, and the autoregulatory mechanisms in a way that require a higher renal perfusion pressure to maintain renal blood flow and filtration to sustain normal excretion of sodium^43^. Furthermore, although GATA5 does not appear to regulate BP through a direct effect on renal sodium handling and excretion, its loss leads to kidney alterations that in turn may contribute to BP increase. Glomerular injuries are usually considered as consequences of increased BP rather than active initiators of the pathogenesis of hypertension^45^. A recent study, however, demonstrated that local glomerular increase in oxidative stress through podocyte-specific overexpression of NOX5 increases BP^46^. The mechanisms by which loss of GATA5 lead to glomerular injuries are not clear, but could involve endothelial cell-mediated podocyte alterations, a key feature in the progression of glomerular lesions^47^,^48^, or secretion of molecules such as BMP4, which is known to promote glomerular injuries^49^. Alteration in the pre-glomerular autoregulatory mechanisms may also contribute to the progression of glomerular injuries in *Gata5*-null mice, since attenuation of these lesions by hydralazine suggests the participation of increased BP in glomerular lesion progression. Renal inflammation, even if secondary, is also a factor that likely sustains BP increase in *Gata5*-null mice^50^. The exact function and mechanisms of action of GATA5 in the kidney warrant further investigations. Be it as it may, our data clearly show that loss of GATA5 from endothelial cells interferes with multiple systems involved in BP regulation.

Low-renin hypertension is a form of hypertension that is particularly frequent in the Afro-American population (up to 52%)^51^. It is characterized by a low-renin plasma activity, salt sensitivity and a high rate of end-organ damage^52^. To our knowledge, the closest mouse model to low-renin hypertension may be the *Npra* (Atrial Natriuretic Protein Receptor A) knockout mice characterized by increased BP and low plasma renin activity, but no salt sensitivity^53^. On the other hand, *Nppa* (which encodes ANP) knockout mice exhibit salt sensitivity, but they are not hypertensive under a regular salt diet^54^. Of note, the *Nppa* system is not altered in *Gata5*-null mice. Both *Nppa* and *Nppb* transcripts level are unchanged in embryonic *Gata5*-null hearts, but are increased in adult hypertrophied hearts^9^. *Npra* transcript levels are also unchanged in *Gata5*-null kidneys. The *Gata5*-null mice exhibit several essential features of low-renin hypertension: significant increase in BP, low-renin plasma activity, salt sensitivity and severe renal alterations. Similar to patients with low-renin hypertension, *Gata5*-null mice respond well to thiazide therapy and may be a useful model to further analyse the pathophysiology and progression of disease.

Although the results from the three consortia CHARGE, GLOBAL BPgen and AGEN-BP represented an important advance in hypertension research, the collective effect of all BP loci identified explains only a small fraction (2%) of BP heritability^5^,^55^,^56^. Additional variants, associated with BP traits remain, therefore, to be identified. Here, we report two common *GATA5* variants that are associated with prescription of anti-hypertensive medication in two independent populations from the ADVANCE^23^ and OHGS^24^ studies, suggesting that *GATA5* might also be involved in BP regulation in humans. Although the variants identified would not affect GATA5 protein sequence (rs6061245 is located within the third intron of *GATA5*, whereas rs6587239 is a synonymous polymorphism in the fifth exon), they might have consequences on GATA5 expression levels. Indeed, with better insight into the factors regulating protein expression, intronic and synonymous single-nucleotide polymorphisms (SNPs) have received increased attention. The functional role of SNPs within introns in altering transcription levels has been clearly validated and linked to several pathologies including hypertension^57^,^58^. Similarly, synonymous SNPs are linked to a plethora of diseases in humans^59^. While the mechanisms involved are not fully elucidated, experimental and computational studies indicate that synonymous SNPs could impact splicing accuracy, translation fidelity, mRNA structure and protein expression/folding by modifying mRNA stability and translational processes^59^.

In conclusion, we have identified *Gata5* as a new gene involved in the regulation of BP. *Gata5*-null mice represent a unique model of hypertension that reproduces several features of human essential hypertension. This model might be especially useful for addressing the pathophysiology of age-related salt sensitivity in hypertension, and for elucidating the mechanisms of low-renin hypertension. Finally, the *Gata5*-null mice provide a new tool for addressing gene–gene and gene–environment interactions in the onset and progression of hypertension and other diseases of the endothelium.

## Methods

### Animals

All the experiments carried on mice were approved by the institutional animal care and use committee (IACUC) of the University of Ottawa under the reference: BMI-1983. Floxed *Gata5* mice^9^ were used and gene inactivation was achieved by crossing with mice harbouring the CRE recombinase under the control of the cytomegalovirus (CMV) promoter (for obtaining *Gata5-null* mice) or the Tie2 or SM22 promoters (for endothelium- or smooth-muscle-specific inactivation, respectively). Both Tie2 and SM22-cre mice are on a C57/B6 background. SM22-Cre mice were obtained from the Jackson Laboratories (stock number 01791). Non-null littermates were used as controls.

### BP measurement

Systolic and diastolic BPs as well as heart pulse were measured by tail-cuff plethysmography in trained conscious mice (3-month-old male and female mice for steady-state experiment on *Gata5*-null mice, and Tie2 and SM22-cre *Gata5*-null mice) using a BP2000 Visitech model. BP was measured every day in the same room at the same hour for 8 consecutive days. The BP measurements presented are the mean of the last 5 consecutive days.

### Echocardiography

Transthoracic echocardiography (Doppler and M-mode) was performed using a visual sonics Vevo 770 ultrasound system with a RMV 707 30-MHz transducer (4-month-old male mice).

### High- and low-salt diet

Acute salt loading: 1% NaCl diluted in drinking water was administered *ad libitum* to mice (3-month-old male mice) for 24 h and urine was collected by metabolic cages.

Chronic high- and low-salt study was performed on 8-month-old male mice: high- (8% NaCl, TD. 92012) and low-salt (0.025% NaCl, TD. 90228) diets were purchased from Harlan Laboratories. BP was measured the week before treatment. High-salt diet was administered for 6 weeks. BP was measured the last week of the high-salt diet. Low-salt diet was administered for 10 days and BP was measured the last week on this diet. At the end of treatment, mice were fed with regular food diet (0.5% NaCl) for 4 weeks, with BP being measured the last week on this diet.

### Hydrochlorothiazide administration

HCTZ was purchased from Sigma-Aldrich (H2910). Two doses were administered to 3-month-old male mice: (1) a low dose, HCTZ was administered in drinking water (700 mg l^−1^) resulting in a dose of 2.8 mg per day, and (2) a high dose, HCTZ was administered in drinking water (700 mg l^−1^) and chow (1.25 mg g^−1^), resulting in a dose of 8 mg per day. Each treatment was administered for 5 days. BP was measured a week before treatment and during all duration of HCTZ administration.

### Hydralazine administration

Hydralazine hydrochloride (Hydra) was purchased from Sigma-Aldrich (H1753). Hydra was diluted in drinking water at the dose of 200 mg l^−1^ and administered for 4 weeks to 3-month-old male mice. Hydra was renewed every 72 h. BP was measured the week before treatment and the last week of treatment.

### Plasma electrolyte measurement and plasma renin activity

Blood was collected from the facial vein (3- and 12-month-old male mice) in heparinized tubes and plasma was isolated by centrifugation (1,000*g*, 15 min). Plasma was stored at −80 °C. Idexx Laboratories (Markham, ON, CA) measured plasma electrolytes concentrations. Plasma renin activity was measured using the fluorometric Renin assay kit from Abcam (Ab138875) using a TF3/TQ3 FRET peptide. Briefly, the fluorescence of TF3 is quenched by TQ3. Upon cleavage into two separate fragments by renin, the fluorescence of TF3 is recovered. The fluorescent signal was measured using a BMG Fluostar Galaxy fluorescence microplate reader at Ex/Em=540/590 nm.

### Urinary electrolyte and aldosterone measurement

Three- and 12-month-old male mice were kept in metabolic cages for 5 consecutive days. Urine was collected every 24 h for the last 3 days. Idexx Laboratories measured plasma electrolytes concentrations. Aldosterone was measured using the Cayman Enzyme immunoassay (10004377).

### Tubules and glomeruli isolation

Individual freehand microdissected proximal tubules, thick ascending limb and cortical collecting ducts were isolated from control mice following a protocol described in ref. 60. Briefly, 3-month-old C57Bl/6 male mice weighing 22–25 g were killed by intraperitoneal injection of ketamine (100 mg kg^−1^) and xylazine (10 mg kg^−1^) for anaesthesia followed by decapitation. The left kidney was quickly removed, and 2–3-mm coronal slices were placed in chilled dissection dishes 8 °C for freehand dissection. The composition of standard dissection medium was as follows: NaCl: 105 mM; NaHCO_3_: 25 mM; Na acetate: 10 mM; NaHPO_4_: 2.3 mM; KCl: 5 mM; CaCl_2_: 1.8 mM; MgSO_4_: 1.0 mM; glucose: 8.3 mM; alanine: 5 mM and 1% albumin. The final osmolality was set at 300 mOsmol kg^−1^. Glomeruli were isolated using magnetic microbeads^61^. Briefly, 3-month-old male mice were anesthetized by intraperitoneal injection of ketamine (100 mg kg^−1^) and xylazine (10 mg kg^−1^) and perfused with 8 × 10^7^ Dynabeads M-450Tosylactivated (Invitrogen, ref 140.13) diluted in 40 ml of phosphate-buffered saline through the heart. The kidneys were removed, minced into 1-mm^3^ pieces and digested in collagenase (1 mg ml− collagenase A—Roche, ref 10103578001- and 100 U ml^−1^ DNAse I—Roche, ref 10104159001—in Hank's balanced salt solution—HBSS) at 37 °C for 30 min with gentle agitation. The collagenase-digested tissue was gently pressed through a 100-μm-cell strainer using a flattened pestle and the cell strainer was then washed with 5 ml of HBSS. The filtered cells were passed through a new cell strainer without pressing and the cell strainer washed with 5 ml of HBSS. The cell suspension was then centrifuged at 200*g* for 5 min. The supernatant was discarded and the cell pellet was resuspended in 2 ml of HBSS. Finally, glomeruli containing Dynabeads were gathered by a magnetic particle concentrator and washed for at least three times with HBSS. During the procedure, kidney tissues were kept at 4 °C except for the collagenase digestion at 37 °C.

### *In vitro* vascular reactivity of isolated arteries

Mesenteric vascular beds were isolated from *Gata5-null* mice and their controls (3-month-old male mice) and placed into a container filled with ice-cold Krebs–Henseleit modified physiological salt solution (in mmol l^−1^: 120 NaCl, 25 NaHCO_3_, 4.7 KCl, 1.18 KH_2_PO_4_, 1.18 MgSO_4_, 2.5 CaCl_2_, 0.026 EDTA and 5.5 glucose). Second-order branches of superior mesenteric artery were dissected and cut into rings of 2 mm in length and were mounted on a multichannel wire myograph (Model 620 M, DMT, Danish Myo Technology, Denmark). Vessel segments were mounted on 25-μm wires in a vessel bath chamber for isometric tension recording. Each chamber contained 5 ml of physiological salt solution bubbled constantly with 95% O_2_ plus 5% CO_2_, 37 °C. At the beginning of each experiment, arteries were contracted by administration of 60 mmol l^−1^ high-K^+^ solution or 10 μmol l^−1^ phenylephrine to test for functional integrity. Endothelium-dependent and −independent relaxations were assessed by measuring isometric force responses to acetylcholine (1 nmol l^−1^ to 10 μmol l^−1^) and Diethylamine NONOate sodium salt hydrate (1 nmol l^−1^ to 10 μmol l^−1^). Contractile responses mediated by phenylephrine (1 nmol l^−1^ to 10 μmol l^−1^) were also evaluated in the arteries.

### Tissue sampling

Hearts, kidneys and mesenteric arteries were harvested from 3–4- and 12-month-old male mice and rinsed in ice-cold PBS. The heart was weighed and cut into two parts (transversal cut). The base was fixed in 4% paraformaldehyde for morphological studies. The apex was kept for mRNA and protein studies. Kidneys were weighted. One kidney was cut transversally into two parts, one for mRNA, and the other for proteins extraction. The second kidney was cut transversally into two parts, one was fixed in 4% formaldehyde and the other was included in tissue-tek and frozen in liquid nitrogen-cooled isopentane for immunofluorescence studies. All non-fixed samples were frozen in liquid nitrogen, and kept at −80 °C.

### Flow cytometry

Immune cells of 3-month-old male mice were isolated from spleen, kidney, bone marrow and thymus by flushing and pelleting (bone marrow) or by mechanical dissociation followed by centrifugation through a percoll gradient (kidney, spleen and thymus). Erythrocytes were lysed with ammonium-chloride-potassium lysing buffer, and the resulting leukocytes were counted on a haemocytometer. Cells were stained for flow cytometry analysis with anti-CD45 (Becton Dickinson, ref: 25-0451-82, dilution: 1/1,000), anti-B220 (eBioscience, ref: 45-0452-80, dilution: 1/1,000), anti-CD11b (eBioscience, ref: 12-0112-82, dilution: 1/1,000), anti-CD11c (eBioscience, ref: 11-0114-85, dilution: 1/1,000), anti-NKp46 (eBioscience, ref: 48-3351-82, dilution: 1/1,000), anti-CD8 (eBioscience, ref: 17-0081-81, dilution: 1/1,000), anti-TCRβ (Becton Dickinson, ref: 563221, dilution: 1/1,000) and Fixable Viability Dye (eBioscience, ref: 65-0865-14). Samples were acquired on CyAnADP 9 flow cytometer (Beckman Coulter) using Summit acquisition software (V). Data were analysed using Kaluza flow analysis software V (Beckman Coulter). An example of this analysis is shown in Supplementary Fig. 12.

### Histology

Tissues (Kidney and hearts) fixed in 4% paraformaldehyde were embedded in paraffin, sectioned at 4-μm intervals, and processed by the uOttawa Histology Core facility. Similarly, all stainings (Periodic acid Schiff, Masson's trichrome staining) were performed by the histology core of the University of Ottawa. Masson's trichrome was used to evaluate fibrosis. Fibrosis area was determined using imageJ (1.43) software.

### Immunofluorescence

Transverse kidney 4-μm sections were stained using primary antibodies against CD45 (Pharmigen, 550539, dilution: 1/500), F4/80 (Abcam Ab6640, dilution: 1/500) or NCC (Millipore, AB3553, dilution: 1/1,000). Briefly, sections were blocked with 5% BSA for 30 min and incubated with the primary antibody overnight at room temperature. After rinsing with PBS, the sections were incubated for 1 h with the appropriate secondary antibody (Donkey anti-rat IGG (H+L) secondary antibody, Alexa Fluor 594 conjugate—Life technologies, ref A-21209, dilution: 1/250—for CD45 and F4/80, and Goat anti-rabbit IGG (H+L) secondary antibody, Alexa Fluor 488 conjugate—Life technologies, ref A-11008, dilution: 1/250- for NCC). Images were acquired blindly to the genotype or treatments with a Zeiss AxioObserver.D1 Microscope mounted with an AxioCam MRm CCD. A minimum of five fields per section of renal cortex was recorded at × 10 and × 20. The positive-stained area was determined using imageJ (1.43) software.

### Scoring of kidney damage

The pathologist of the Kidney Research Center (Ottawa Hospital) acquired images of kidney sections at × 40 with a Zeiss AxioObserver A1microscope mounted with an Olympus DP73 digital colour camera and analysed them (blind to the genotype or treatment of mice). A semi-quantitative score was used to evaluate the degree of damage^62^. A minimum of 50 glomeruli in each group was examined and the severity of the lesion was graded from 0 to +3: a +1 lesion represented an involvement of 25% of the glomerulus, +2 of 25–50%, while a +3 lesion indicated that 50–75% of the glomerulus was involved. Observation for other pathological lesions included glomerular collapse (as absent, partial or total); glomerular hypertrophy (absent or present); distribution of glomerulosclerosis (absent, diffuse widespread in the cortex, superficial focal, located in the inner or outer half of the cortex and focal juxta-medullar.

### Human primary endothelial cells

Human primary endothelial cells were purchased from Promocell: Human Coronary artery endothelial cells (ref C-14022, Lot: 91001010.7P); Human Cardiac Microvascular Endothelial Cells (ref C-14029, Lot: 20224401P); Human Pulmonary Microvascular Endothelial Cells (ref C-14027, Lot: 9090301P) and HDMECs (ref C-14016, Lot: 4060603P).

### Generation of *GATA5* knockdown HDMEC cell line

*GATA5* MISSION TRC2 pLKO.shRNA (TRCN0000431556) and MISSION TRC2 pLKO-puro Non-Mammalian shRNA Control (SHC202) plasmids were purchased from Sigma-Aldrich. Viral particles were generated by co-transfection of phoenix cells with the lentiviral vector (*GATA5* TRC2 shRNA or the shRNA control plasmids), the packaging vector psPAX2 and the envelope vector pMD2G. Co-transfection was performed using the Qiagen's Effectene transfection reagent (301425). Twenty-four hours after transfection, medium was discarded and replaced by Endothelial Growth Medium 2 (Promocell, C-22021). After 48 h, the medium (EGM2) was collected, filtered and used for infection of HDMECs (Promocell, C-14016). Infected cells were selected with addition of puromycin (Sigma-Aldrich, P8833) to culture medium at the concentration of 2.5 μg ml^−1^ for 15 days. Control (HDMEC-pLKO-Ctrl) and HDMEC-GATA5-KD cells were then grown and maintained in EGM2 containing puromycin at the concentration of 0.25 μg ml^−1^.

### Microarray analysis

mRNA from HDMEC-GATA5-KD cells and their controls (HDMEC-pLKO-Ctrl) was extracted using Qiagen's RNEASY mini-kit (74104) following the manufacturer recommendations. mRNA quality and integrity were assessed with Fragment analyser (Advanced Analytical), and samples with RQN>9 were retained for analysis (three of each group). StemCore Laboratories at Ottawa Institute Research Hospital performed samples preparation and hybridization on Affymetrix Human Gene 2.0 ST arrays (902136). Non-used mRNA was used for qPCR. Fold-change analysis was performed using the R package limma, *P* values were Benjamini-Hochberg (BH) adjusted. HuGene chip annotations are based on the HuGene-2_0-st-v1.na35.hg19.transcript.csv annotations downloaded from Affymetrix. The first gene identifier associated with a given probe set (transcript cluster identifier) was chosen as the symbol representing it. The pathway functional analysis was performed through the use of Qiagen's Ingenuity Pathway Analysis (IPA, Qiagen, Redwood City, www.qiagen.com/ingenuity).

Microarray data have been deposited in the Gene Expression Omnibus database (NCBI) under accession code: GSE71838.

### QPCR

Frozen tissues (kidneys, heart and mesenteric arteries) were homogenized in TRIzol (Life Technologies, 15596018) using FastPrep beads (MP-Bio, 6913-100). Due to the small amount of mRNA expected from mesenteric arteries, once separated in the aqueous phase, mRNA was then extracted using RNeasy micro-kit (Qiagen, 74004). cDNAs were generated using the Omniscript RT kit (Qiagen, 205113), and qPCR was performed as previously described. Briefly, transcript levels were analysed in a RotorGene 6000 apparatus (Corbett Life Science). The reactions were performed in duplicate for each sample using the RotorGene SYBR Green PCR Kit (Qiagen, 204076). To normalize gene expression, we used the geometric mean of multiple internal reference genes Ct (*RS16*, *Ubc*, *Hprt* and *Gapdh* for mice experiments, *GAPDH* and *UBC* for human cell experiments) as described by Vandesompele *et al*.^63^ Values in control conditions were set as 1 for each gene. The sequences of the specific primers are detailed in Supplementary Table 3.

### Western blot

Endothelial cells (HDMEC-GATA5-KD and HDMEC-pLCKO-Ctrl), mesenteric arteries and kidney proteins were extracted in SDS 1% buffer containing PhosSTOP phosphatases inhibitor (Roche, 04906845001) and cOmplete protease inhibitor (Roche, 04693116001) reagents. Protein concentration was estimated according to the Pierce method. Membranes were incubated overnight (4 °C) with the respective antibodies: GATA5 (Santa Cruz, Sc-47600, dilution: 1/1,000), phospho-NOS3-ser1177 (Cell Signalling, ref 9571, dilution: 1/2,000), phospho-NOS3-thr495 (Cell Signalling, ref 9574, dilution: 1/500), NOS3 (Abcam, ref Ab66127, dilution: 1/1,000), phospho-Akt-ser473 (Cell Signalling, ref 9271, dilution: 1/1,000), phospho-Akt-thr308 (Cell Signalling, ref 4056, dilution: 1/1,000), Akt (Cell Signalling, ref 9272, dilution: 1/1,000), phospho-PTEN-ser380 (Cell Signalling, ref 9551, dilution: 1/2,500), PTEN (Cell Signalling, ref 9188, dilution: 1/1,000), phospho-PKD1-ser241 (Cell Signalling, ref 3061, dilution: 1/2,500), PKD1 (Cell Signalling, ref 3062, dilution: 1/1,000), phospho-(Ser/Thr) PKA substrate antibody (Cell Signalling, ref 9621, dilution: 1/1,000), NCC (Millipore, AB3553), αENaC (Pierce, PA1–920A, dilution: 1/1,000), NKCC2 (Abcam, Ab171747, dilution: 1/1,000), α1-Na/K ATPase subunit (Abcam, Ab7671, dilution: 1/10,000) and β1-Na/K ATPase subunit (SantaCruz Biotechnologies, Sc-25709, dilution: 1/1,000). Human GATA5 was detected with an antibody generated by the laboratory (dilution: 1/1,000)^9^. Chemiluminescent signal was produced using ECL+ solution and detected with a LAS-4000 luminescent image analyzer (General Electric). Relative densitometry was determined using the computer software ImageQuant TL (General Electric). Actin (Santa Cruz Biotechnology, ref Sc-1616) was used as a protein-loading control. Full scans of western blots are available in Supplementary Fig. 9.

### Nitrotyrosine measurement

Mesenteric arteries proteins were extracted in SDS 0.25% buffer containing PhosSTOP phosphatases inhibitor (Roche, 04906845001) and cOmplete protease inhibitor (Roche, 04693116001) reagents. Protein concentration was estimated according to the Pierce method. Nitrotyrosine content was measured with the 3-Nitrotyrosine ELISA Kit (ab116691) from Abcam according to the manufacturer's recommandations. Colour development was measured at 600 nm with a powerwave XS2 microplaque spectrophotometer (Biotek, Winooski, USA).

### Human association studies

OHGS (cohort 1) is a case–control study aimed at discovering genetic variants that modify the risk of CAD, independent of diabetes. Study participants were recruited from the angiography clinic of the University of Ottawa Heart Institute or from the Ottawa Community for Asymptomatic Controls. CAD cases were defined as having at least one coronary artery with >50% stenosis and controls were asymptomatic of CAD or among angiographic samples, had minimal (<30%) coronary artery stenosis. Diabetics were excluded from participating in the study. More detailed information on the CAD definitions and recruitment procedures of the OHGS have been detailed previously. Supplementary Table 4 details the clinical characteristics of patients taking or not hypertensive medication^23^,^64^. The OHGS samples were genotyped on Affymetrix 500 K or 6.0 GWAS SNP genotyping arrays. Genotyped variants 56 kb either side of the *GATA5* gene (within the genomic coordinates of chr20:60,982,420–61,107,159) were tested for their association with hypertensive medication use.

The ADVANCE (cohort 2) trial is a factorial, multi-center, randomized controlled clinical of 11,140 participants recruited from 215 centres in 20 countries assessing the effect of treatment with hypertension and glucose-lowering drugs on incident diabetic complications^5^,^24^. All subjects were type-2 diabetic patients, and inclusion criteria into ADVANCE were the following: age of 55 years and older and diagnosis of type-2 diabetes at age of 30 years or older and one of the following: (1) a history of major macrovascular disease, or (2) history of major microvascular disease, or (3) diagnosis of type-2 diabetes over 10 years before entry in study, or (4) presence of another major risk factor for vascular disease (including smoking, dyslipidemia or microalbuminuria), or (5) age of 65 years or older. All subjects were ascertained at entry into the study for a variety of macro- and microvascular diabetes associated phenotypes including hypertension, defined as being treated for hypertension; coronary heart disease symptoms, including myocardial Infarction, stroke, transient ischaemic attack, angina and major adverse coronary events; and for chronic kidney disease, associated phenotypes including glomerular filtration rate and urinary albumin creatinine ratio. A subset of 2301 ADVANCE subjects was genotyped using the Affymetrix Genome-Wide Human SNP Arrays 5.0 or 6 and passed quality controls. They were used for further association analysis. Supplementary Table 5 details the clinical characteristics of those taking or not hypertension medications in the ADVANCE GWAS study.

### *In silico* analysis

Human Splicing Finder is an online tool to predict the effects of mutations on splicing signals or to identify splicing motifs in any human sequence^25^. It contains all available matrices for auxiliary sequence prediction. Consequences of *GATA5* variant rs6587239 on SRSs were analysed by comparative analysis of motifs present on *GATA5* fifth exon with either an adenine or a guanine (ancestral) on position 27.

### Statistics

The results are reported as mean±s.e.m. Statistical analysis was performed using Graphpad V5 software. The *in vivo* experiments were performed blind to the genotype and, when possible, to the experimental conditions. The data were analysed blind to the genotype and the experimental conditions. Sample size was determined empirically based on pilot analyses. All inclusion and exclusion criteria were pre-established according to the IACUC guidelines. The Kolmogorov–Smirnov (when 5≤*n*<7) or the d' Agostino–Pearson test (when *n*≥7 per group) was used to analyse normality. Two-group comparisons were performed using one tailed *t*-test if normality was confirmed; otherwise the Mann–Whitney test was used. The Mann–Whitney test was also used for statistical analysis of experiment with *n*<5 per group. ANOVA was used for multivariate comparisons followed by Bonferonni correction for multiple comparisons. HCTZ and salt-sensitivity experiments were analysed by a repeated measures two-factor ANOVA test followed by Bonferonni correction for multiple comparisons. Vascular reactivity dose responses were analysed by comparison of best-fit values (effector concentration for half-maximum response and Hill slope) using an F-test. For GWAS studies, Frequentist association tests for additive model were performed using SNPTEST^49^ (version 2.5). SNPTEST implements logistic regression analysis for discrete variables and linear regression analysis for continuous variables. R (version 3.1.2) was used for data handling, and SAS Enterprise Guide (version 4.3) was used to create the clinical characteristics tables. Differences with *P*<0.05 are considered significant.

## Additional information

**How to cite this article:** Messaoudi, S. *et al*. Endothelial Gata5 transcription factor regulates blood pressure. *Nat. Commun.* 6:8835 doi: 10.1038/ncomms9835 (2015).

## Supplementary Material

Supplementary Information Supplementary Figures 1-12 and Supplementary Tables 1-5

## Figures and Tables

**Table 1:** Logistic regression analysis results for HTN in the OHGS (*n*=5,835) and ADVANCE (*n*=2,301) studies.

SNP	MA	MAF			Additive model		
		All	HTN	Non-HTN	Beta	s.e.	*P*
*OHGS*							
rs6587239	T	0.487	0.497	0.479	0.0725	0.0368	0.0492
rs6061245	T	0.499	0.488	0.508	−0.0809	0.0374	0.0307
							
*ADVANCE*							
rs6587239	T	0.484	0.510	0.460	0.2037	0.0595	0.0006
rs6061245	T	0.496	0.473	0.517	−0.1879	0.0610	0.0020

ADVANCE, Action in Diabetes and Vascular Disease: Peterax and Diamicron MR Controlled Evaluation; HTN, patients with anti-hypertensive medication; MA, minor allele; MAF, minor allele frequency; Non-HTN, patients without anti-hypertensive medication; OHGS, Ottawa Heart Genomics Study; SNP, single-nucleotide polymorphism.

Crude betas are given for the minor allele. Results are corrected for coronary artery disease status in the OHGS study. MAFs are given for all tested individuals, those who have been prescribed anti-hypertensive medication (HTN) and those without prescription of anti-hypertensive medication (non-HTN). Results are calculated assuming an additive model.
