# Supplementary material for: Endothelial Gata5 transcription factor regulates blood pressure
Source: Nat Commun. 2015 Nov 30;6:8835. doi: 10.1038/ncomms9835 (PMC4696516; doi:10.1038/ncomms9835)
Supplement: Supplementary Information — Supplementary Figures 1-12 and Supplementary Tables 1-5 [file ncomms9835-s1.pdf]

SUPPLEMENTARY FIGURES

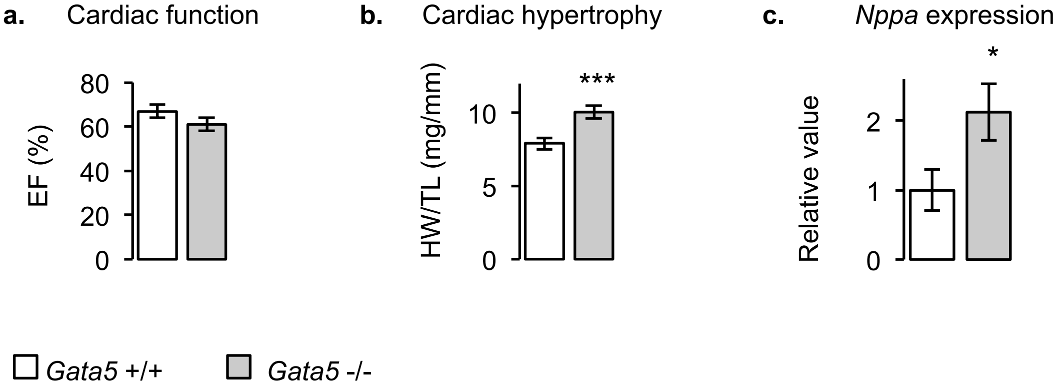

Supplementary Figure 1: Loss of *Gata5* in mice induces a hypertensive-like cardiac phenotype
a. Cardiac function: no difference in ejection fraction between the *Gata5*-null mice and *Gata5* +/+ mice was measured by echocardiography (n=6/group). The results are reported as mean±SEM (T-test).
b. Cardiac hypertrophy: The *Gata5*-null mice have larger hearts in comparison with control littermates as assessed by the heart weight to tibia length (HW/TL) ratio (n=4-6/group). The results are reported as mean ±SEM. *** $p<0.005$ *vs Gata5* +/+ mice (Mann-Whitney test).
c. Molecular remodeling: The mRNA expression of the molecular remodeling marker (*Nppa:* atrial natriuretic protein A) is altered in the *Gata5*-null mice in comparison with their control littermates. (n=4-6/group). The results are reported as mean±SEM. * $p<0.05$ *vs Gata5* +/+ mice (Mann-Whitney test).

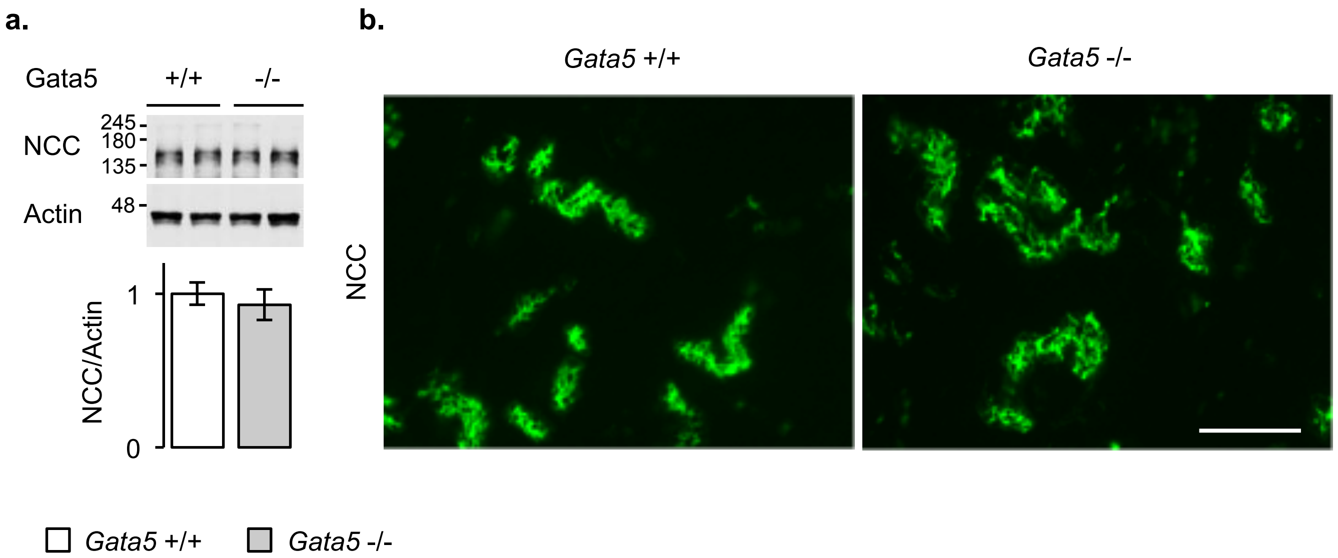

Supplementary Figure 2: Loss of *Gata5* in mice has no effect on renal expression of sodium chloride transporter NCC

a. Quantification of the sodium chloride transporter NCC in the kidney of *Gata5*-null mice and their controls. There are no differences as assessed by western blot (n=4/group). The results are reported as mean±SEM. **b.** Immunostaining of NCC in the kidney of *Gata5*-null mice and *Gata5* +/+ mice (Mann-Whitney test) (Scale bar= 100 µm).

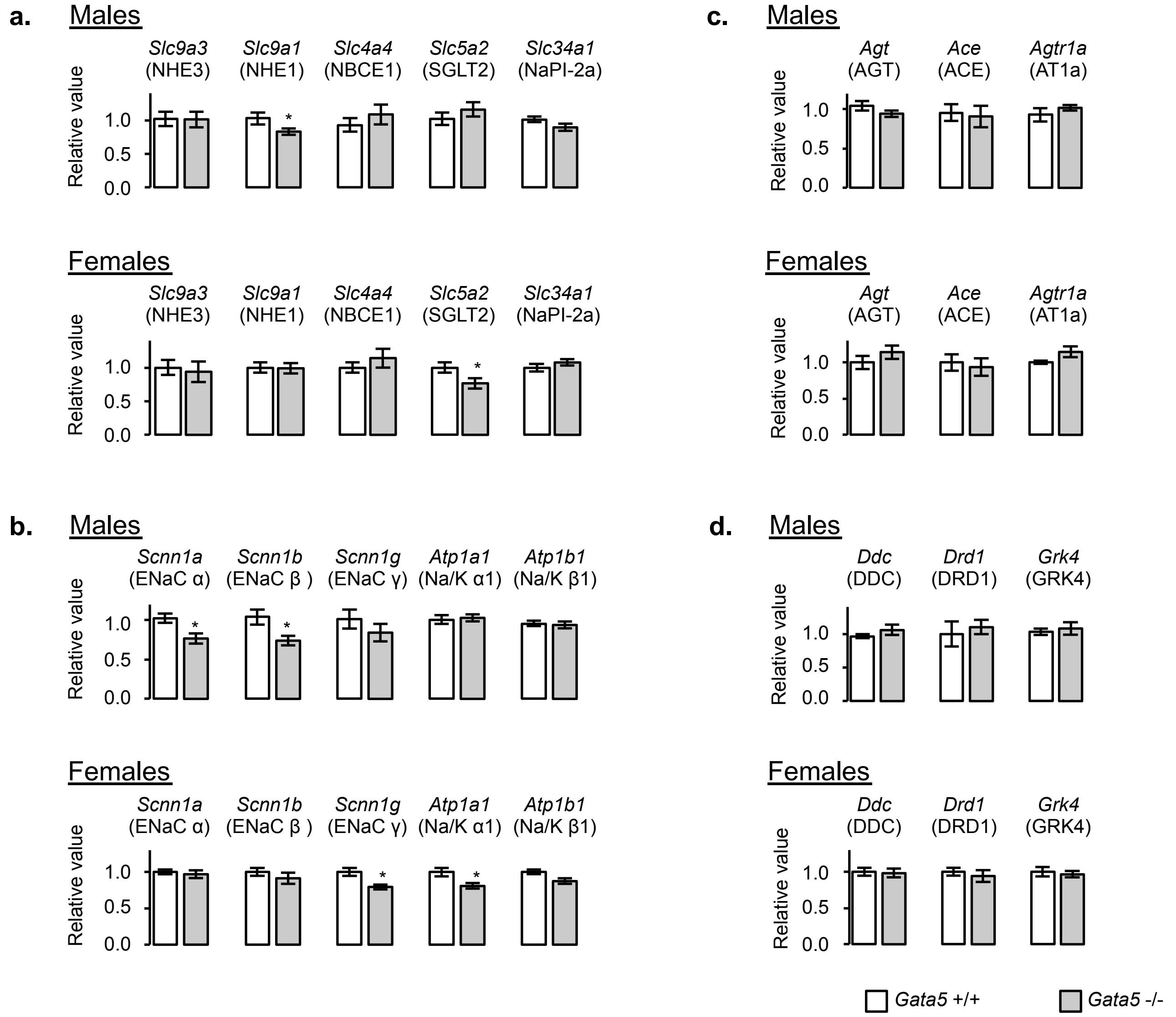

Supplementary Figure 3: Quantitative-PCR analysis of kidney gene expression

a. Expression of proximal tubule sodium transporter in *Gata5*-null mice and their controls. *Slc9a3:* sodium/ hydrogen exchanger 3 (NHE3); *Slc3a1*: sodium/hydrogen exchanger 1 (NHE1); *Slc4a4*: Electrogenic sodium bicarbonate cotransporter 1 (NBCE1); *Slc5a2*: sodium/glucose cotransporter 2 (SGLT2); *Slc34a1*: Sodium/ phosphate cotransporter (NaPI-2a). (males: n=6/group; females: n=7-6/ group). The results are reported as mean±SEM. * p<0.05 *vs Gata5* +/+ mice (T-test).

b. Expression of distal nephron sodium transporter in *Gata5*-null mice and their controls. *Scnn1a*: epithelial sodium channel α subunit (EnaCα); *Scnn1b*: epithelial sodium channel β subunit (EnaCβ); *Scnn1g*: epithelial sodium channel γ subunit (EnaCγ); *Atp1a1*: sodium/potassium ATPase α1 subunit (Na/K ATPase α1); *Atp1b1*: sodium/potassium ATPase β1 subunit (Na/K ATPase β1) (males: n=6/group; females: n=7-6/ group) The results are reported as mean±SEM. * p<0.05 *vs Gata5* +/+ mice (T-test).

c. Expression of genes of the intra-renal renin angiotension system in the kidney of *Gata5*-null mice and their controls. *Agt*: Angiotensinogen (AGT); *Ace*: angiotensin converting enzyme (ACE), *Agtr1a*: Angiotensin receptor type 1a (AT1a). (males: n=6/group; females: n=7-6/ group). The results are reported as mean±SEM (T-test).

d. Expression of genes of the intra-renal dopaminergic system in the kidney of *Gata5*-null mice and their controls. *Ddc*: Dopa decarboxylase (DDC); *Drd1*: Dopamine receptor D1 (DRD1); *Grk4:* G protein-coupled receptor kinase 4 (GRK4). (males: n=6/group; females: n=7-6/ group). The results are reported as mean ±SEM (T-test).

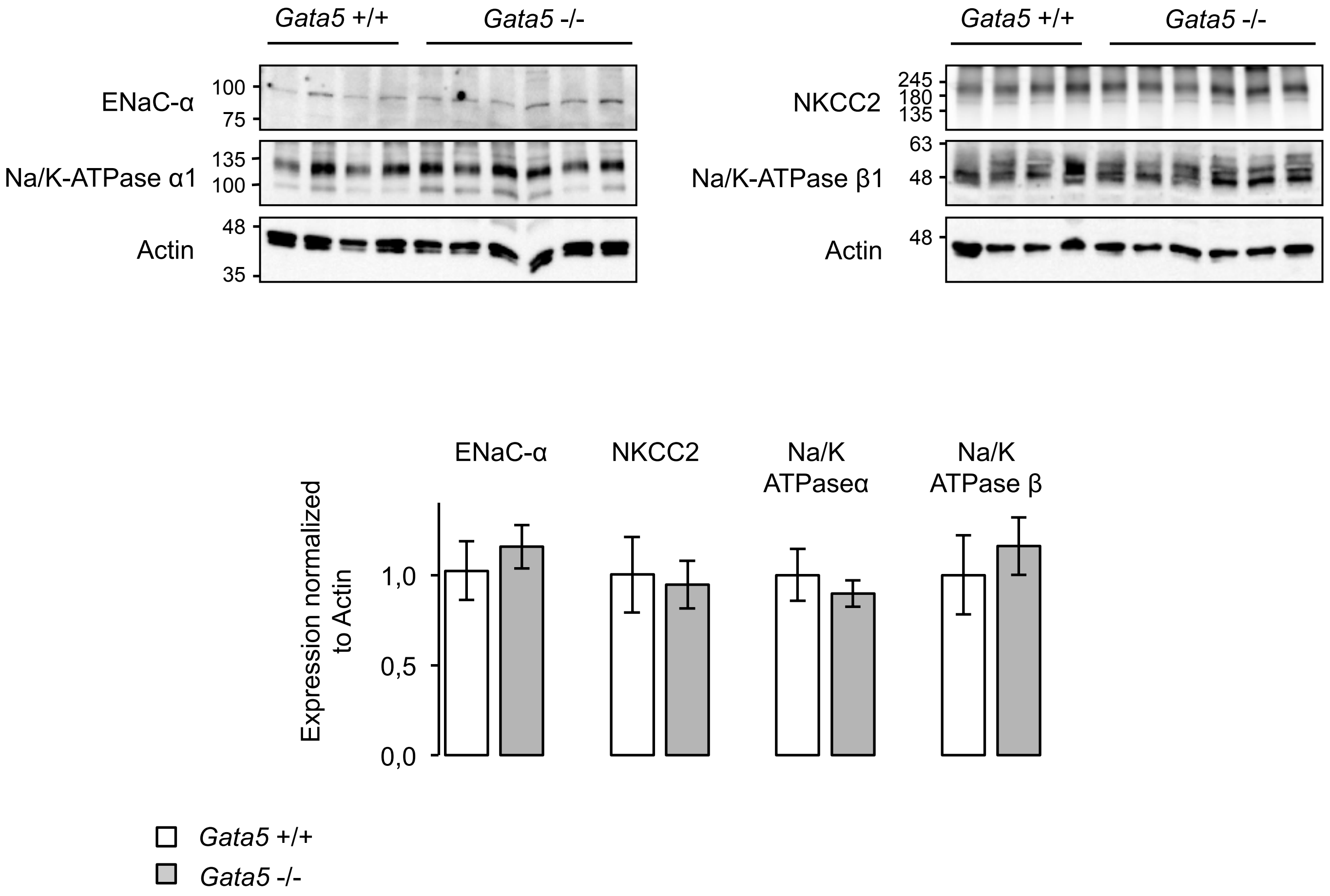

Supplementary Figure 4: Protein expression of sodium channels and transporters in the kidney
Western blots of renal sodium channels and transporters in *Gata5*-null mice and their controls. ENaCα: epithelial sodium channel α subunit; NKCC2: Sodium-Potassium-Chloride co-transporter 2; Na/K ATPase α1: sodium/potassium ATPase α1 subunit; Na/K ATPase β1: sodium/potassium ATPase β1 subunit (n=4-6/group). The results are reported as mean±SEM (Mann-Whitney test).

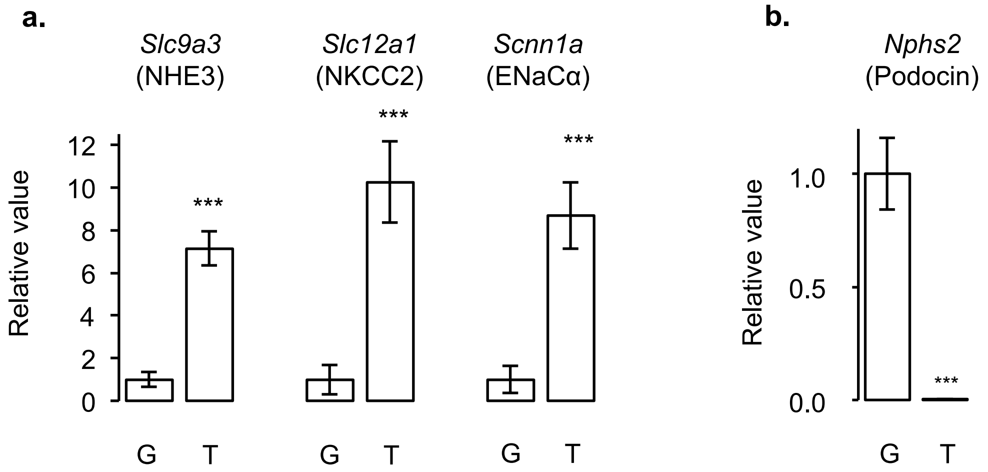

T: tubules

G: Glomeruli

Supplementary Figure 5: Quantitative PCR with glomerular (G) and tubules (T) markers

Tubules and Glomeruli were prepared from Wt mice kidneys.

a. All the segments of the tubules were present in our preparations as assessed by the presence of the sodium/hydrogen exchanger 3 gene (*Slc9a3* codes for NHE3; expressed mostly in proximal tubule), the sodium/potassium/chloride cotransporter 2 gene (*Slc12a1* codes for NKCC2; expressed mostly in Henle Loop), and the epithelial sodium channel α subunit gene (*Scnn1a* codes for ENaCα; expressed mostly in distal tubule). (n=3-5/group). The results are reported as mean±SEM. *** p<0.001 *vs* Glomeruli (Mann-Whitney test).

b. Podocin gene (*Nphs2*) was used as a marker of glomeruli. (n=3-5/group). The results are reported as mean±SEM. *** p<0.001 *vs* Glomeruli (Mann-Whitney test).

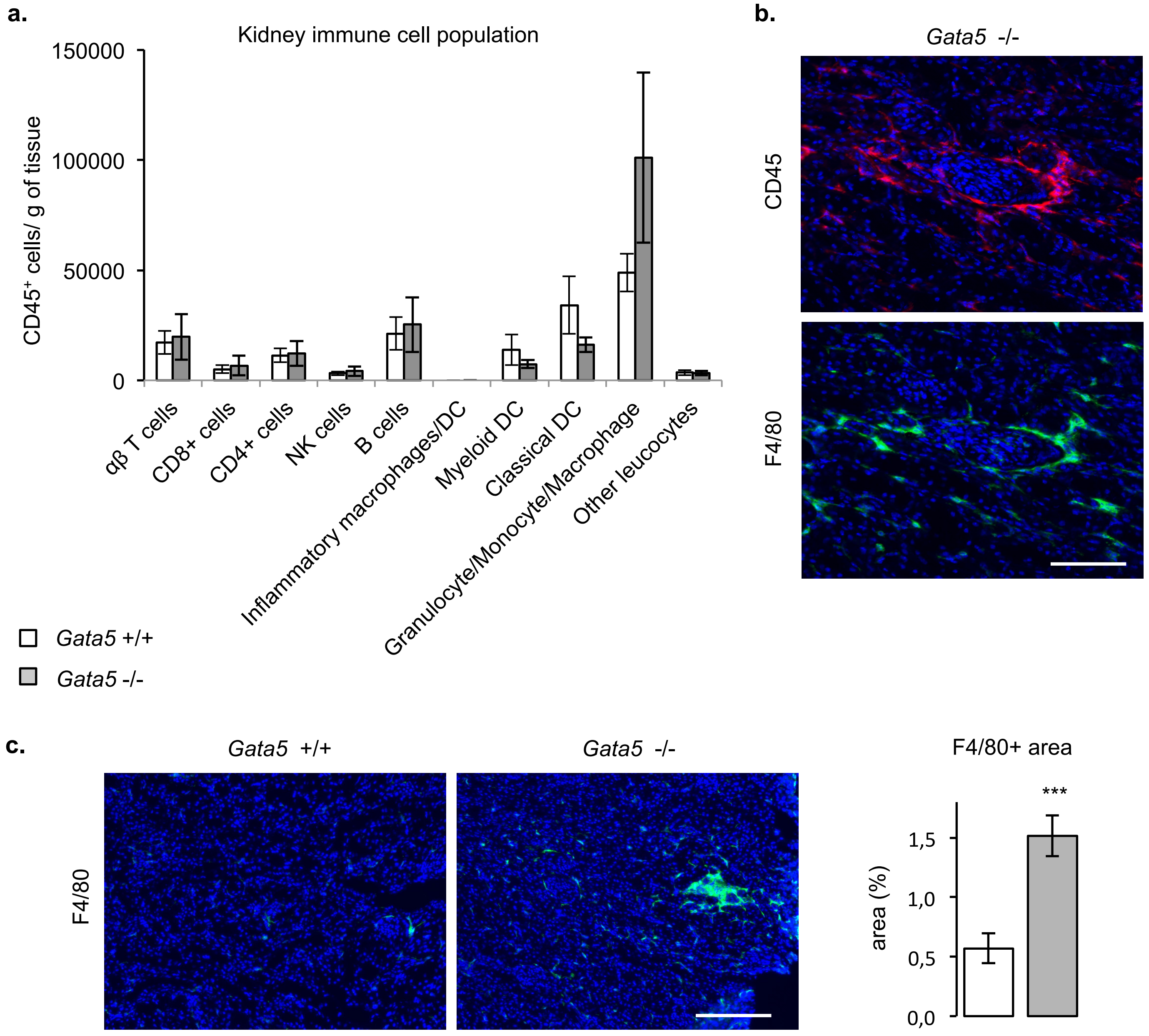

Supplementary Figure 6: Identification of the CD45+ cells in the kidney of *Gata5*-Null mice

a. Identification of the major immune cell populations in the kidney of Gata5-null mice and their controls through determination of the relative proportion of all CD45+ leukocyte subsets based on their expression of TCRβ, NKp46, CD8, B220, CD11b, and CD11c by flow cytometry analysis. The trend toward increased myeloid population in the kidney suggests a macrophage infiltration. (n=4-6/group) (Mann-Whitney test).

b. Immunostaing of CD45 (upper panel, red) and F4/80 (macrophages, lower panel, green) on *Gata5*-null mice kidney serial cut (8 um thick) confirmed that most of CD45+ infiltrated cells were macrophages (Scale bar= 100 µm),

c. Immunostaining (left) and quantification (right) of F4/80+ cell infiltration in the kidney. (n=4-6/group). The results are reported as mean±SEM. *** *p*<0.001 *vs Gata5* +/+ mice (Mann-Whitney test) (Scale bar= 200 µm).

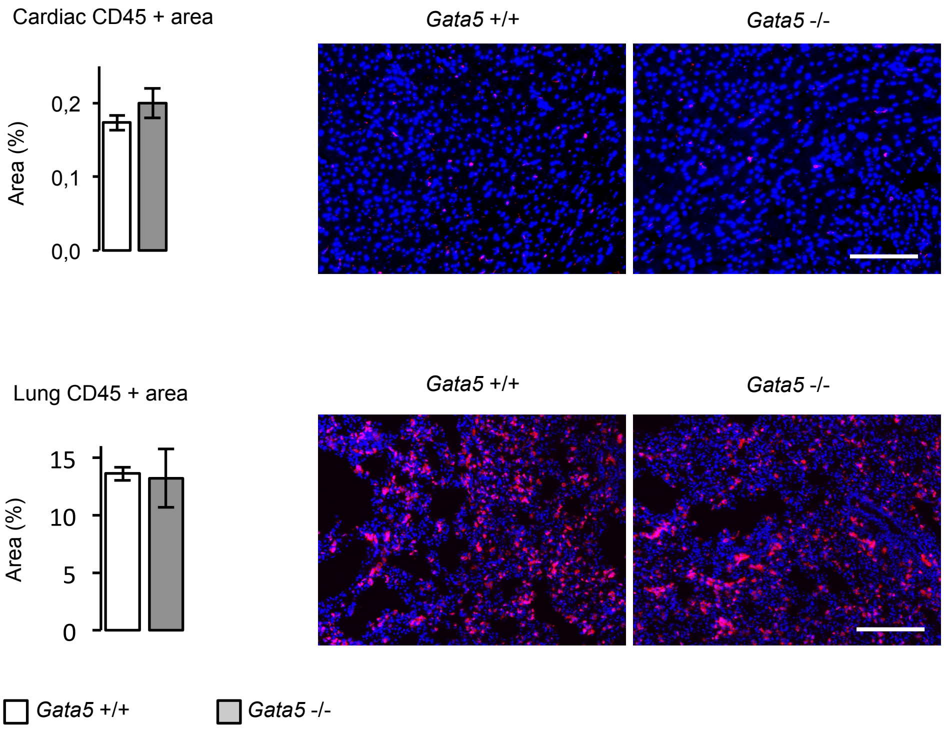

Supplementary Figure 7: Quantification of CD45+ cells infiltration in the heart and lung of *Gata5*-null mice and their controls

Immunostaing and quantification of CD45+ cells in the heart and lung of *Gata5*-null mice and their controls. No changes were found. (n=5/group). The results are reported as mean±SEM (Mann-Whitney test). (Scale bar= 200 µm).

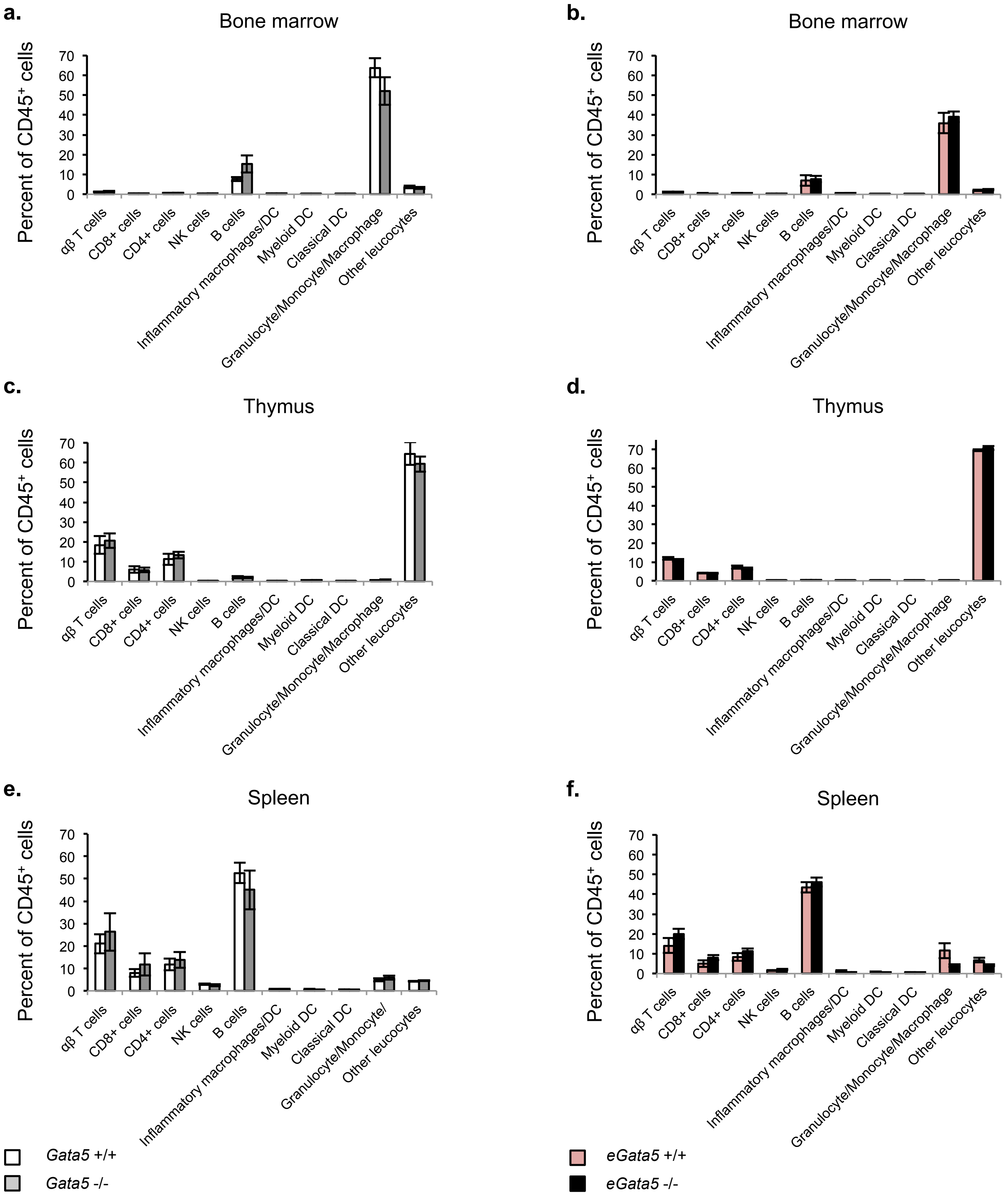

Supplementary Figure 8: Analysis total and endothelial-specific *Gata5*-null mice immune systems by flow cytometry

Live leukocyte were isolated from the bone marrow, spleen and thymus of total and endothelial specific *Gata5*-null mice (*Gata5*: a, c and e; e*Gata5:* b, d and f*)* and their controls and were analyzed based on their expression of CD45, TCRβ, NKp46, CD8, B220, CD11b, and CD11c. The relative proportions of all CD45+ live leukocytes were determined for each group. No differences were found (Mann-Whitney test). (*Gata5*: n=4-6/group; *eGata5*: n: 5-7/group)

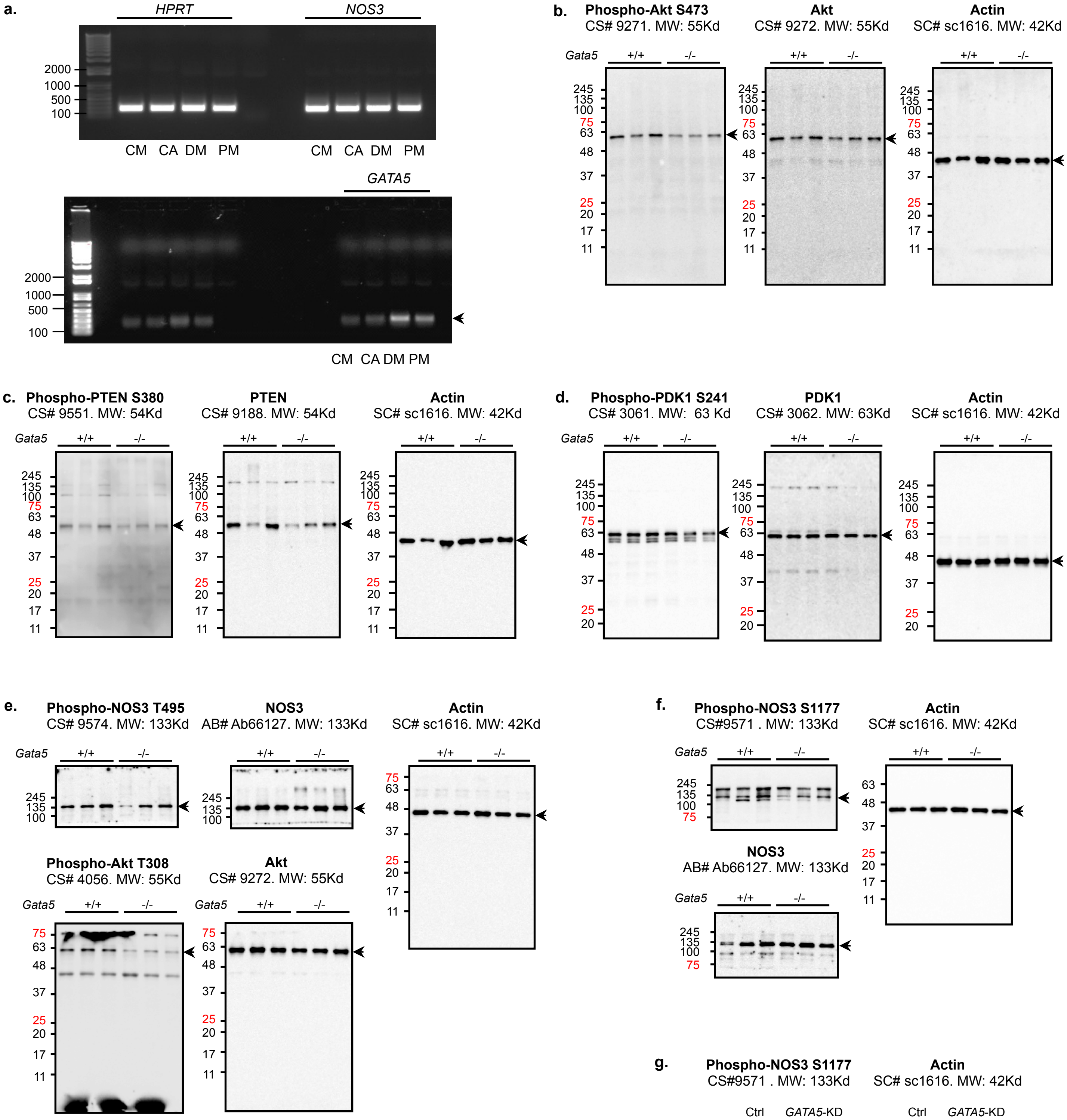

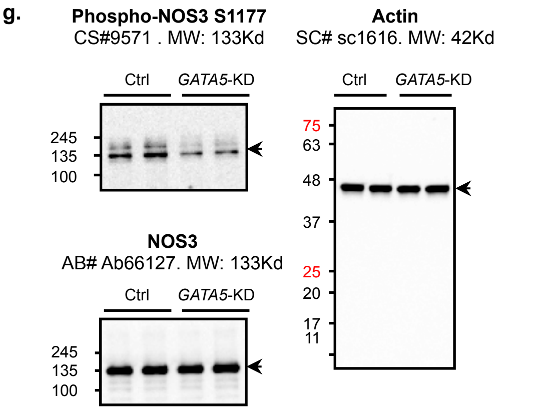

Supplementary figure 9: Uncropped gels from representative PCR gel and western blots shown in Figures 3 and 4.

a. PCR gels corresponding to panel (a) in Figure 3.

b-f. Blots corresponding to panel (h) in Figure 3. Corresponding loading control and non-phosphorylated proteins for each blot are also shown. Actin blot shown in Fig 3h is (b), NOS3 blot shown in Fig 3h is (f), and Akt blot shown in Fig 3h is (b).

g. Blots corresponding to panel (e) in Figure 4.

CS: Cell Signalling; SC: SantaCruz Biotechnologies; AB: Abcam.

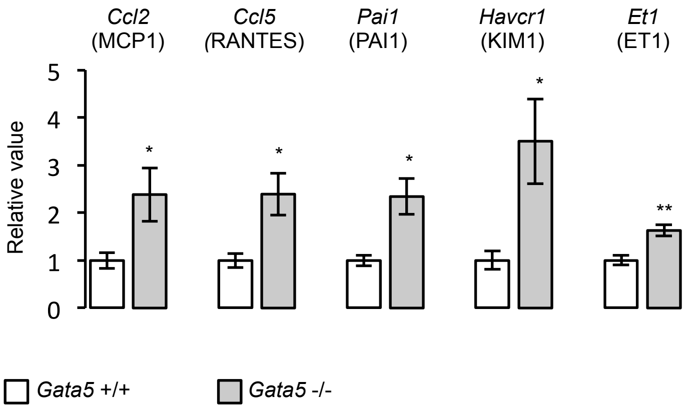

Supplementary Figure 10: Loss of *Gata5* increases the expression of several pro-inflammatory markers in old mice (300 days)

qPCR analysis of inflammation and kidney injury markers in 12 months-old male *Gata5*-Null mice and their controls . *Ccl2*: macrophage chemo-attractant protein 1 (MCP1); *Ccl5* : chemokine Ligand 5 (RANTES); *Pai1*: Plasminogen activator inhibitor 1 (PAI1); *Havcr1*: kidney injury molecule (KIM-1), *Et1*: endothelin 1 (ET1). (n=5-7/group). The results are reported as mean±SEM. * p<0.05 *vs Gata5* +/+ mice; ** p<0.05 *vs Gata5* +/+ mice (Mann-Whitney test for *Havcr1*, T-test for *Ccl2*, *Ccl5*, *Pai1* and *Et1*).

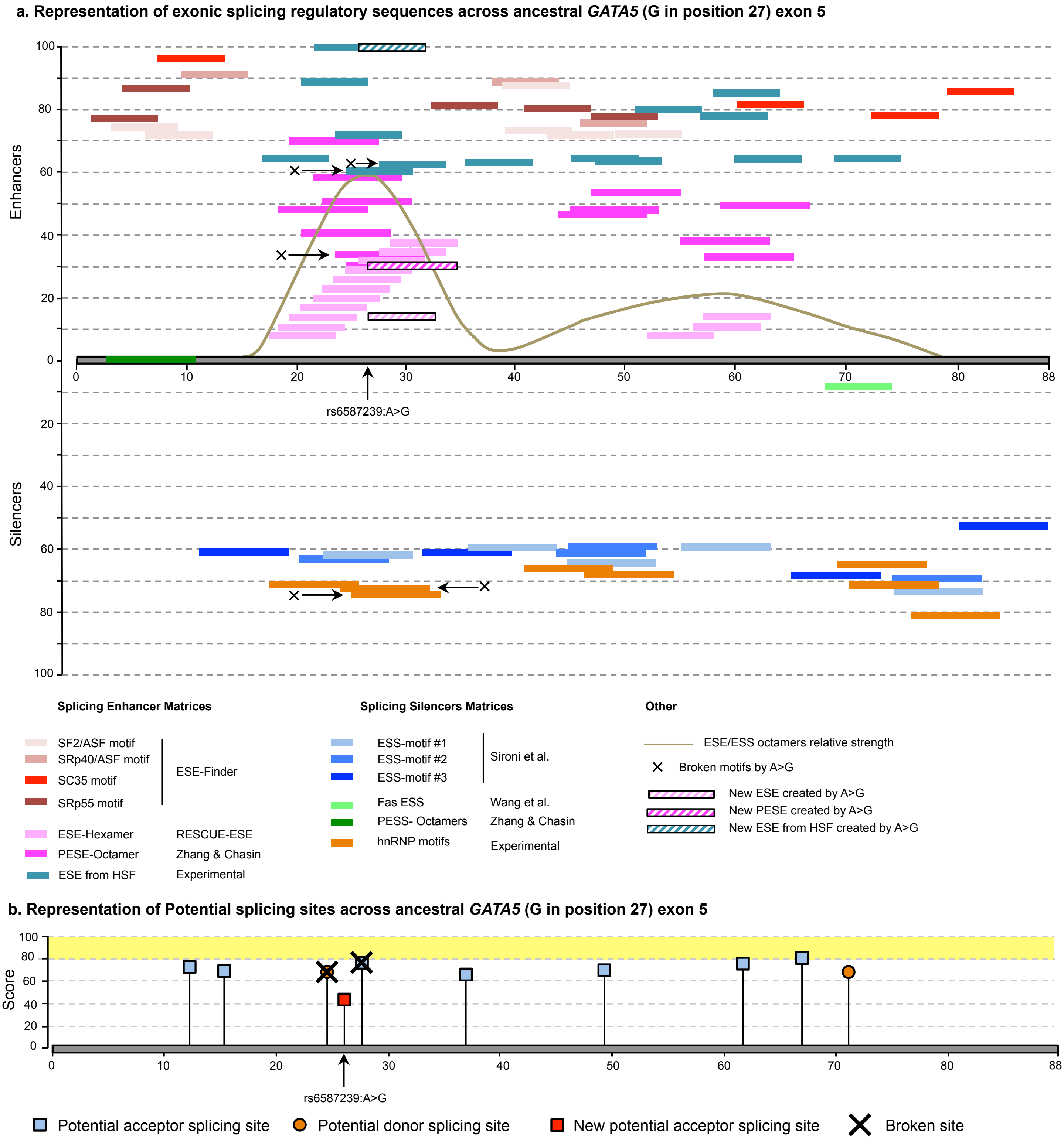

Supplementary Figure 11: ***In silico* analysis of variant rs6587239 consequences on splicing regulatory sites**

Human *GATA5* Exon5 (88 bp) was analyzed with Human Splicing Finder software V3. HSF search for splicing regulatory sequences with both exact motif approach or degenerated consensus motif approach. HSF integrates all available published matrices as well as home-made algorithms to identify exonic and intronic regulatory sequences and calculate consensus values (y axis) and the thresholds that determine whether a sequence is considered as a motif or not. rs6587239 (A>G) is located within a region rich in regulatory sequences. Substitution of G by A alters motifs and creates new ones with potential alteration of splicing. ESE: Exonic Splicing Enhancer; PESE: Putative Exonic Splicing Enhancer; ESS: Exonic Splicing Silencer; PESS: Putative Exonic splicing enhancer

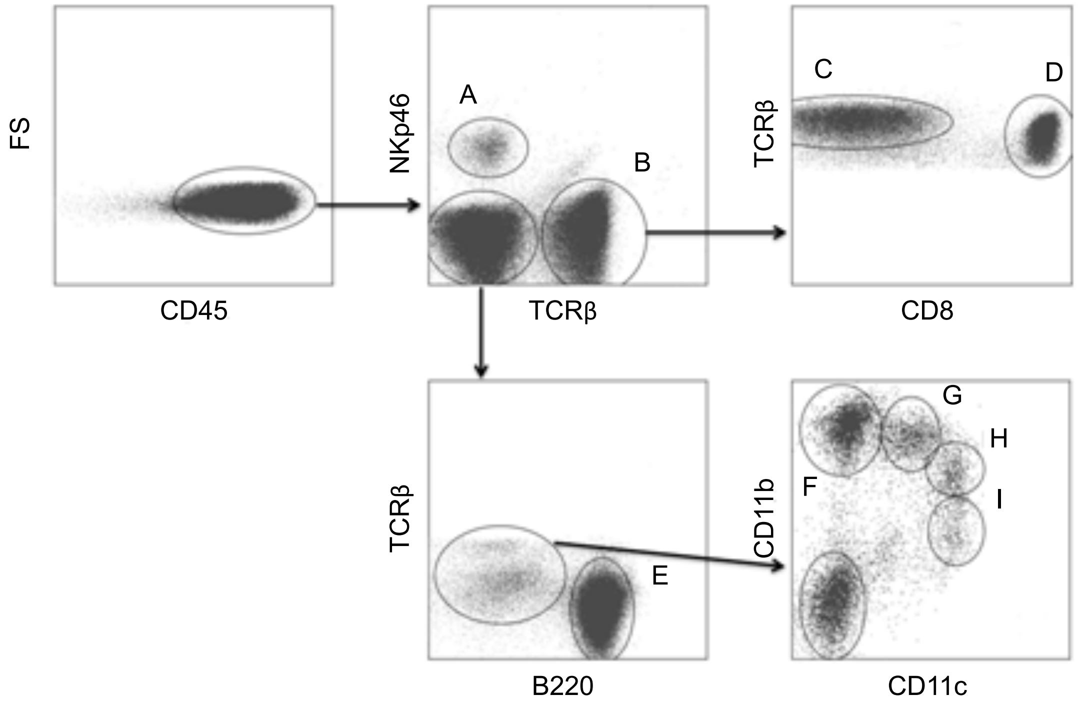

Gate	Definition	Principle Cell Type
A	$CD45^{+}$ $TCR\beta^{-}$ $NKp46^{+}$	NK cells
B	$CD45^{+}$ $TCR\beta^{-}$ $NKp46^{-}$	T Cells
C	$CD45^{+}$ $TCR\beta^{-}$ $NKp46^{-}$ $CD8^{-}$	CD4+ T cells
D	$CD45^{+}$ $TCR\beta^{-}$ $NKp46^{-}$ $CD8^{+}$	CD8+ T cells
E	$CD45^{+}$ $TCR\beta^{-}$ $NKp46^{-}$ $B220^{+}$	B cells
F	$CD45^{+}$ $TCR\beta^{-}$ $NKp46^{-}$ $B220^{-}$ $CD11b^{hi}$ $CD11c^{-}$	Granulocytes/Monocytes/Macrophages
G	$CD45^{+}$ $TCR\beta^{-}$ $NKp46^{-}$ $B220^{-}$ $CD11b^{hi}$ $CD11c^{Low}$	Inflammatory macrophages/DC
H	$CD45^{+}$ $TCR\beta^{-}$ $NKp46^{-}$ $B220^{-}$ $CD11b^{hi}$ $CD11c^{High}$	Myeloid dendritic cells
I	$CD45^{+}$ $TCR\beta^{-}$ $NKp46^{-}$ $B220^{-}$ $CD11b^{Low}$ $CD11c^{High}$	Classical dendritic cells
J	$CD45^{+}$ $TCR\beta^{-}$ $NKp46^{-}$ $B220^{-}$ $CD11b^{-}$ $CD11c^{-}$	Other leucocytes

Supplementary Figure 12: Representative illustration of the gating strategy for immune cell subsets identification by flow cytometry analysis

Live leukocytes were analyzed based on their expression of CD45, TCRβ, NKp46, CD8, B220, CD11b, and CD11c. An example of this analysis for the spleen is shown. The identities of each population analyzed, as well as the principle immune cell type contained within that population, is given on the table above (the illustration represents an analysis on the spleen of a Ctrl mouse)

SUPPLEMENTARY TABLES

Supplementary Table1. Electrolyte balance in control and *Gata5*-null mice

**Age**	**3 months**				**12 months**	
**Diet**	***Regular salt***		***High salt (24h)***		***Regular salt***	
**Genotype**	***Gata5*** $^{+/+}$	***Gata5*** $^{-/-}$	***Gata5*** $^{+/+}$	***Gata5*** $^{-/-}$	***Gata5*** $^{+/+}$	***Gata5*** $^{-/-}$
Food/water metabolism (n)	13	13	9	9	5	7
Water intake (ml/24h)	3.96± 0.19	4.14±0.12	4.97± 0.42$^{\#\#}$	5.58±0.49$^{\#\#}$	3.67± 0.22	4.23±0.46
Urine excretion (ml/24h)	1.17± 0.11	1.04± 0.09	1.86± 0.26$^{\#\#}$	2.00± 0.24$^{\#\#}$	0.80± 0.11	0.87± 0.09
Food intake (g/24h)	3.98± 0.23	4.15±0.12	4.13± 0.10	4.10±0.09	3.32± 0.24	3.33±0.15
Feces (g/24h) [A]	1.58± 0.06	1.66±0.07	1.50± 0.09	1.48±0.09	1.05± 0.12	1.15±0.12
Na^+ intake (mmol/24h)	0.34± 0.02	0.36± 0.01	1.20± 0.07$^{\#\#}$	1.31± 0.08$^{\#\#}$	0.28± 0.02	0.29± 0.01
Urine (n)	6	6	6	6	5	7
Na^+ excretion (mmol/24h)	0.18± 0.02	0.16± 0.01	0.74± 0.07$^{\#\#}$	0.69± 0.07$^{\#\#}$	0.11± 0.02	0.12± 0.02
K^+ excretion (mmol/24h)	0.37± 0.03	0.31± 0.02	0.48± 0.04 $^{\#\#}$	0.46± 0.04$^{\#\#}$	0.22± 0.03	0.27± 0.03
Cl^- excretion (mmol/24h)	0.37± 0.02	0.34± 0.01	0.96± 0.06$^{\#\#}$	0.93± 0.08$^{\#\#}$	0.22± 0.03	0.27± 0.04
Ur Na^+ excretion/ Na^+ intake	0.47± 0.04	0.43± 0.04	0.56± 0.03$^{\#\#}$	0.54± 0.04$^{\#\#}$	0.38± 0.04	0.42± 0.05
Plasma (n)	6	6			5	7
Na^+ concentration (mMol)	147.71± 0.68	145.71± 0.78	-	-	145.14± 0.55	145.14± 0.55
K^+ concentration (mMol)	4.61± 0.17	4.84± 0.12	-	-	5.93± 0.25	5.93± 0.25
Cl^-concentration (mMol)	109.43± 0.97	108.43± 1.29	-	-	108.86± 0.51	108.86± 0.51
Hematocrit (%)	46.7± 0.1	45.1± 0.5	-	-	46.4± 0.4	43.8± 0.7

A- The n for feces measurement in 3 months-old mice is (9). $^{\#\#}$ <0.01 *vs* untreated mice (t-test).

Supplementary Table 2. Differentially expressed genes between HDMEC-GATA5-KD cells and their controls (HDMEC-pLKO-Ctrl)

Reg	FC	P.Value	Gene symbol	Description
Up	3.62	0.0084	B3GALT2	UDP-Gal:betaGlcNAc beta 1,3-galactosyltransferase, polypeptide 2
Up	3.33	0.0082	EPGN	epithelial mitogen
Up	3.28	0.0063	SULT1E1	sulfotransferase family 1E, estrogen-preferring, member 1
Up	2.97	0.0314	GS1-600G8.5	
Up	2.85	0.0205	LOC101060632	
Up	2.83	0.0154	OARD1	O-acyl-ADP-ribose deacylase 1
Up	2.69	0.0096	GANC	glucosidase, alpha; neutral C
Up	2.67	0.0124	OR1L8	olfactory receptor, family 1, subfamily L, member 8
Up	2.62	0.0044	IGFBP5	insulin-like growth factor binding protein 5
Up	2.50	0.0107	CLDN1	claudin 1
Up	2.50	0.0123	VCAN	versican
Up	2.45	0.0449	RNU7-124P	RNA, U7 small nuclear 124 pseudogene
Up	2.45	0.0096	MIR4324	microRNA 4324
Up	2.44	0.0044	PLAT	plasminogen activator, tissue
Up	2.36	0.0044	GS1-124K5.3	
Up	2.36	0.0049	SERPINE2	serpin peptidase inhibitor, clade E (nexin, plasminogen activator inhibitor type 1), member 2
Up	2.36	0.0176	RP11-3L8.3	
Up	2.36	0.0082	COL1A2	collagen, type I, alpha 2
Up	2.24	0.0214	MT-TD	mitochondrially encoded tRNA aspartic acid
Up	2.23	0.0095	BMP4	bone morphogenetic protein 4
Up	2.21	0.0366	RNU7-80P	RNA, U7 small nuclear 80 pseudogene
Up	2.20	0.0063	ITGB4	integrin, beta 4
Up	2.19	0.0063	PCDH17	protocadherin 17
Up	2.17	0.0208	LINC01021	long intergenic non-protein coding RNA 1021
Up	2.15	0.0096	PKP2	plakophilin 2
Up	2.15	0.0073	POLR2J	polymerase (RNA) II (DNA directed) polypeptide J, 13,3kDa
Up	2.15	0.0107	FAT1	FAT atypical cadherin 1
Up	2.14	0.0189	PRKXP1	protein kinase, X-linked, pseudogene 1
Up	2.13	0.0235	RP11-88I18.2	
Up	2.09	0.0177	ITGB8	integrin, beta 8
Up	2.08	0.0074	CD34	CD34 molecule
Up	2.06	0.0096	LINC00984	long intergenic non-protein coding RNA 984
Up	2.06	0.0145	SULF1	sulfatase 1
Up	2.04	0.0168	SERPINB7	serpin peptidase inhibitor, clade B (ovalbumin), member 7
Up	2.02	0.0096	LINC00607	long intergenic non-protein coding RNA 607
Up	2.00	0.0126	ABCA1	ATP-binding cassette, sub-family A (ABC1), member 1
Up	2.00	0.0096	PPP1R3C	protein phosphatase 1, regulatory subunit 3C
Up	1.99	0.0122	LRRC71	leucine rich repeat containing 71
Up	1.99	0.0096	SDC2	syndecan 2
Up	1.97	0.0095	PTCHD4	patched domain containing 4

Supplementary Table 2

Up	1.96	0.0107	TMC7	transmembrane channel-like 7
Up	1.95	0.0088	TNFRSF10D	tumor necrosis factor receptor superfamily, member 10d, decoy with truncated death domain
Up	1.95	0.0289	LOC100188947	
Up	1.94	0.0214	MFAP3	microfibrillar-associated protein 3
Up	1.91	0.0108	SLC4A7	solute carrier family 4, sodium bicarbonate cotransporter, member 7
Up	1.91	0.0092	PVR	poliovirus receptor
Up	1.90	0.0096	NPFF	neuropeptide FF-amide peptide precursor
Up	1.90	0.0149	SNORD113-8	small nucleolar RNA, C/D box 113-8
Up	1.89	0.0096	NPEPPS	aminopeptidase puromycin sensitive
Up	1.88	0.0399	ITGA11	integrin, alpha 11
Up	1.87	0.0124	LINC01061	long intergenic non-protein coding RNA 1061
Up	1.87	0.0295	SNORA32	small nucleolar RNA, H/ACA box 32
Up	1.86	0.0179	LINC00607	long intergenic non-protein coding RNA 607
Up	1.86	0.0131	CPA4	carboxypeptidase A4
Up	1.86	0.0222	GFRA1	GDNF family receptor alpha 1
Up	1.85	0.0252	LINC00152	long intergenic non-protein coding RNA 152
Up	1.84	0.0208	MIR31HG	MIR31 host gene
Up	1.84	0.0101	RP11-841O20.2	
Up	1.84	0.0095	TULP3	tubby like protein 3
Up	1.83	0.0272	LOC102724529	
Up	1.83	0.0201	SRSF1	serine/arginine-rich splicing factor 1
Up	1.83	0.0192	PRRT1	proline-rich transmembrane protein 1
Up	1.83	0.0111	UGCG	UDP-glucose ceramide glucosyltransferase
Up	1.83	0.0229	ST7	suppression of tumorigenicity 7
Up	1.83	0.0485	MIR1827	microRNA 1827
Up	1.82	0.0101	SLC39A6	solute carrier family 39 (zinc transporter), member 6
Up	1.82	0.0096	LINC00607	long intergenic non-protein coding RNA 607
Up	1.82	0.0175	SLC2A12	solute carrier family 2 (facilitated glucose transporter), member 12
Up	1.81	0.0184	LOC644794	
Up	1.81	0.0244	LOC101929463	
Up	1.81	0.0391	CEACAM19	carcinoembryonic antigen-related cell adhesion molecule 19
Up	1.80	0.0498	RAP2C-AS1	RAP2C antisense RNA 1
Up	1.80	0.0111	SLC35D2	solute carrier family 35 (UDP-GlcNAc/UDP-glucose transporter), member D2
Up	1.80	0.0290	C8orf4	chromosome 8 open reading frame 4
Up	1.78	0.0407	MIR612	microRNA 612
Up	1.77	0.0133	KLF2	Kruppel-like factor 2
Up	1.76	0.0288	HSPB6	heat shock protein, alpha-crystallin-related, B6
Up	1.76	0.0222	MIR4653	microRNA 4653
Up	1.76	0.0270	LOC101927345	
Up	1.75	0.0096	TBCK	TBC1 domain containing kinase
Up	1.75	0.0160	ROR1	receptor tyrosine kinase-like orphan receptor 1
Up	1.74	0.0466	LINC00665	long intergenic non-protein coding RNA 665
Up	1.74	0.0189	SOX9	SRY (sex determining region Y)-box 9
Up	1.74	0.0096	PLXNA1	plexin A1

Supplementary Table 2

Up	1.74	0.0152	COA5	cytochrome c oxidase assembly factor 5
Up	1.74	0.0096	LMLN	leishmanolysin-like (metallopeptidase M8 family)
Up	1.74	0.0170	PTPRM	protein tyrosine phosphatase, receptor type, M
Up	1.74	0.0198	C10orf111	chromosome 10 open reading frame 111
Up	1.73	0.0112	ATXN7L3B	ataxin 7-like 3B
Up	1.73	0.0214	HTR1D	5-hydroxytryptamine (serotonin) receptor 1D, G protein-coupled
Up	1.73	0.0223	KCNRG	potassium channel regulator
Up	1.73	0.0184	IGF2BP1	insulin-like growth factor 2 mRNA binding protein 1
Up	1.73	0.0310	LOC440434	
Up	1.72	0.0248	LINC00665	long intergenic non-protein coding RNA 665
Up	1.71	0.0172	TSPAN14	tetraspanin 14
Up	1.71	0.0214	LINC00520	long intergenic non-protein coding RNA 520
Up	1.70	0.0362	ZNF807	zinc finger protein 807
Up	1.70	0.0244	MBTD1	mbt domain containing 1
Up	1.70	0.0133	LINC00641	long intergenic non-protein coding RNA 641
Up	1.70	0.0310	LOC101929038	
Up	1.70	0.0108	ITPR3	inositol 1,4,5-trisphosphate receptor, type 3
Up	1.70	0.0152	LINC00665	long intergenic non-protein coding RNA 665
Up	1.69	0.0154	LYSMD1	LysM, putative peptidoglycan-binding, domain containing 1
Up	1.69	0.0118	ANGPTL4	angiopoietin-like 4
Up	1.69	0.0364	LINC01000	long intergenic non-protein coding RNA 1000
Up	1.69	0.0364	LINC01000	long intergenic non-protein coding RNA 1000
Up	1.69	0.0172	VCAN-AS1	VCAN antisense RNA 1
Up	1.69	0.0361	LINC01000	long intergenic non-protein coding RNA 1000
Up	1.68	0.0192	LOC730101	
Up	1.68	0.0169	LRRC32	leucine rich repeat containing 32
Up	1.68	0.0477	LOC102724440	
Up	1.67	0.0149	MIR548V	microRNA 548v
Up	1.67	0.0124	DICER1	dicer 1, ribonuclease type III
Up	1.67	0.0214	CELF2	CUGBP, Elav-like family member 2
Up	1.67	0.0178	RAB40B	RAB40B, member RAS oncogene family
Up	1.67	0.0269	RNU6-616P	RNA, U6 small nuclear 616, pseudogene
Up	1.66	0.0230	OSTM1	osteopetrosis associated transmembrane protein 1
Up	1.66	0.0146	RNU1-83P	RNA, U1 small nuclear 83, pseudogene
Up	1.66	0.0123	TM4SF18	transmembrane 4 L six family member 18
Up	1.66	0.0133	LOC100129034	
Up	1.66	0.0179	PEAR1	platelet endothelial aggregation receptor 1
Up	1.65	0.0436	LINC01000	long intergenic non-protein coding RNA 1000
Up	1.65	0.0122	TM2D1	TM2 domain containing 1
Up	1.65	0.0480	FAM106CP	family with sequence similarity 106, member C, pseudogene
Up	1.65	0.0172	ARHGAP23P1	Rho GTPase activating protein 23 pseudogene 1
Up	1.65	0.0190	FAM73A	family with sequence similarity 73, member A
Up	1.65	0.0333	RP11-504P24.2	
Up	1.65	0.0234	STYXL1	serine/threonine/tyrosine interacting-like 1
Up	1.65	0.0205	KRT18P49	keratin 18 pseudogene 49

Supplementary Table 2

Up	1.64	0.0176	CHAC1	ChaC glutathione-specific gamma-glutamylcyclotransferase 1
Up	1.64	0.0477	MIR154	microRNA 154
Up	1.63	0.0366	LINC00345	long intergenic non-protein coding RNA 345
Up	1.63	0.0184	RP11-1166P10.1	
Up	1.63	0.0422	hsa-mir-4763	
Up	1.63	0.0252	TUSC2	tumor suppressor candidate 2
Up	1.62	0.0172	ARHGAP23	Rho GTPase activating protein 23
Up	1.62	0.0269	IL6	interleukin 6
Up	1.62	0.0149	CCDC80	coiled-coil domain containing 80
Up	1.62	0.0475	LOC100133331	
Up	1.62	0.0201	HYAL1	hyaluronoglucosaminidase 1
Up	1.62	0.0313	RP11-74E22.6	
Up	1.61	0.0192	ATP13A3	ATPase type 13A3
Up	1.61	0.0161	TSPYL2	TSPY-like 2
Up	1.61	0.0310	LOC100132062	
Up	1.61	0.0176	MGP	matrix Gla protein
Up	1.60	0.0295	LOC729737	
Up	1.60	0.0318	SLC26A2	solute carrier family 26 (anion exchanger), member 2
Up	1.60	0.0152	LAMC2	laminin, gamma 2
Up	1.60	0.0214	ACAD10	acyl-CoA dehydrogenase family, member 10
Up	1.60	0.0190	DIRAS3	DIRAS family, GTP-binding RAS-like 3
Up	1.60	0.0399	HCFC1R1	host cell factor C1 regulator 1 (XPO1 dependent)
Up	1.59	0.0170	CTD-2033D15.1	
Up	1.59	0.0284	HERC2P9	hect domain and RLD 2 pseudogene 9
Up	1.59	0.0189	ANKRD13B	ankyrin repeat domain 13B
Up	1.59	0.0360	LOC101928706	
Up	1.59	0.0366	FOXG1	forkhead box G1
Up	1.59	0.0222	OAF	out at first homolog
Up	1.59	0.0208	COL6A1	collagen, type VI, alpha 1
Up	1.58	0.0389	IGF2BP3	insulin-like growth factor 2 mRNA binding protein 3
Up	1.58	0.0144	EIF4E2	eukaryotic translation initiation factor 4E family member 2
Up	1.58	0.0295	PKD1P5	polycystic kidney disease 1 (autosomal dominant) pseudogene 5
Up	1.58	0.0152	RNU6-945P	RNA, U6 small nuclear 945, pseudogene
Up	1.58	0.0369	MIR516A1	microRNA 516a-1
Up	1.58	0.0295	LOC102725126	
Up	1.58	0.0423	RP11-410L14.2	
Up	1.58	0.0167	OTUD4	OTU deubiquitinase 4
Up	1.58	0.0150	LINC00607	long intergenic non-protein coding RNA 607
Up	1.58	0.0149	LOX	lysyl oxidase
Up	1.57	0.0153	PKD1	polycystic kidney disease 1 (autosomal dominant)
Up	1.57	0.0230	ARPC3	actin related protein 2/3 complex, subunit 3, 21kDa
Up	1.57	0.0399	AGAP5	ArfGAP with GTPase domain, ankyrin repeat and PH domain 5
Up	1.57	0.0449	XYLB	xylulokinase homolog (H, influenzae)
Up	1.57	0.0428	THAP6	THAP domain containing 6

Supplementary Table 2

Up	1.57	0.0394	REC8	REC8 meiotic recombination protein
Up	1.57	0.0409	RPPH1	ribonuclease P RNA component H1
Up	1.56	0.0440	C11orf91	chromosome 11 open reading frame 91
Up	1.56	0.0416	NEK7	NIMA-related kinase 7
Up	1.56	0.0301	FBXW7	F-box and WD repeat domain containing 7, E3 ubiquitin protein ligase
Up	1.56	0.0149	ANKRD52	ankyrin repeat domain 52
Up	1.56	0.0154	PLSCR4	phospholipid scramblase 4
Up	1.56	0.0465	MN1	meningioma (disrupted in balanced translocation) 1
Up	1.56	0.0309	LINC01278	long intergenic non-protein coding RNA 1278
Up	1.55	0.0152	COLGALT1	collagen beta(1-O)galactosyltransferase 1
Up	1.55	0.0189	RNU1-38P	RNA, U1 small nuclear 38, pseudogene
Up	1.55	0.0189	RNU1-38P	RNA, U1 small nuclear 38, pseudogene
Up	1.55	0.0288	LOC100131564	
Up	1.55	0.0348	RNU6-1266P	RNA, U6 small nuclear 1266, pseudogene
Up	1.55	0.0189	SNORD71	small nucleolar RNA, C/D box 71
Up	1.55	0.0214	AGAP6	ArfGAP with GTPase domain, ankyrin repeat and PH domain 6
Up	1.55	0.0309	HBEGF	heparin-binding EGF-like growth factor
Up	1.54	0.0179	ZNF250	zinc finger protein 250
Up	1.54	0.0233	MYEOV	myeloma overexpressed
Up	1.54	0.0184	TNFSF18	tumor necrosis factor (ligand) superfamily, member 18
Up	1.54	0.0175	ZNF514	zinc finger protein 514
Up	1.54	0.0245	LOC101927345	
Up	1.53	0.0340	USP40	ubiquitin specific peptidase 40
Up	1.53	0.0245	ARHGAP23P1	Rho GTPase activating protein 23 pseudogene 1
Up	1.53	0.0227	PODXL	podocalyxin-like
Up	1.53	0.0214	MIDN	midnolin
Up	1.53	0.0444	FAM35A	family with sequence similarity 35, member A
Up	1.53	0.0170	FLNC	filamin C, gamma
Up	1.53	0.0466	PKD1	polycystic kidney disease 1 (autosomal dominant)
Up	1.53	0.0425	AC010974.3	
Up	1.53	0.0349	SECISBP2	SECIS binding protein 2
Up	1.53	0.0466	ZBTB20	zinc finger and BTB domain containing 20
Up	1.52	0.0248	MMP24-AS1	MMP24 antisense RNA 1
Up	1.52	0.0324	RP11-217B7.2	
Up	1.52	0.0366	PNN	pinin, desmosome associated protein
Up	1.52	0.0225	ICAM1	intercellular adhesion molecule 1
Up	1.52	0.0256	ITPR1-AS1	ITPR1 antisense RNA 1 (head to head)
Up	1.52	0.0223	RHBDF1	rhomboid 5 homolog 1 (Drosophila)
Up	1.52	0.0308	DYNC1H1	dynein, cytoplasmic 1, heavy chain 1
Up	1.52	0.0196	ADA	adenosine deaminase
Up	1.52	0.0366	BTG2	BTG family, member 2
Up	1.52	0.0288	ZNF738	zinc finger protein 738
Up	1.52	0.0301	GABRE	gamma-aminobutyric acid (GABA) A receptor, epsilon
Up	1.52	0.0448	NPR2	natriuretic peptide receptor 2
Up	1.51	0.0399	BACE1-AS	BACE1 antisense RNA

Supplementary Table 2

Up	1.51	0.0192	AC010967.2	
Up	1.51	0.0340	FRS2	fibroblast growth factor receptor substrate 2
Up	1.51	0.0447	MID1IP1-AS1	MID1IP1 antisense RNA 1
Up	1.51	0.0205	RP11-521B24.5	
Up	1.50	0.0170	MAMDC2	MAM domain containing 2
Up	1.50	0.0243	SESN2	sestrin 2
Up	1.50	0.0282	TBC1D2	TBC1 domain family, member 2
Up	1.50	0.0251	RP4-791M13.3	
Up	1.50	0.0485	LINC01000	long intergenic non-protein coding RNA 1000
Up	1.50	0.0244	LINC00152	long intergenic non-protein coding RNA 152
Up	1.50	0.0360	LOC101927365	
Up	1.50	0.0406	PELI1	pellino E3 ubiquitin protein ligase 1
Up	1.50	0.0366	LMAN2L	lectin, mannose-binding 2-like
Down	-5.85	0.0043	TRBC2	T cell receptor beta constant 2
Down	-5.75	0.0025	CYP2S1	cytochrome P450, family 2, subfamily S, polypeptide 1
Down	-4.72	0.0044	HTR1B	5-hydroxytryptamine (serotonin) receptor 1B, G protein-coupled
Down	-4.00	0.0044	RP1-66C13.3	
Down	-3.96	0.0044	RP1-66C13.3	
Down	-3.58	0.0118	CTSS	cathepsin S
Down	-3.40	0.0044	MMRN1	multimerin 1
Down	-3.37	0.0095	TMEM156	transmembrane protein 156
Down	-3.34	0.0084	PTPN22	protein tyrosine phosphatase, non-receptor type 22 (lymphoid)
Down	-3.17	0.0133	FABP4	fatty acid binding protein 4, adipocyte
Down	-3.16	0.0149	LOC101927880	
Down	-3.09	0.0050	POMK	protein-O-mannose kinase
Down	-3.08	0.0064	BCHE	butyrylcholinesterase
Down	-3.06	0.0044	MRC1	mannose receptor, C type 1
Down	-3.04	0.0044	MRC1	mannose receptor, C type 1
Down	-3.03	0.0044	CNDP2	CNDP dipeptidase 2 (metallopeptidase M20 family)
Down	-3.02	0.0096	LGALS9B	lectin, galactoside-binding, soluble, 9B
Down	-2.96	0.0497	MX1	MX dynamin-like GTPase 1
Down	-2.92	0.0044	OXCT1	3-oxoacid CoA transferase 1
Down	-2.91	0.0068	CLEC1A	C-type lectin domain family 1, member A
Down	-2.91	0.0179	IFI44	interferon-induced protein 44
Down	-2.89	0.0063	FAM129A	family with sequence similarity 129, member A
Down	-2.82	0.0044	LAMP3	lysosomal-associated membrane protein 3
Down	-2.81	0.0108	CXCL11	chemokine (C-X-C motif) ligand 11
Down	-2.77	0.0096	PREX2	phosphatidylinositol-3,4,5-trisphosphate-dependent Rac exchange factor 2
Down	-2.76	0.0079	CISD2	CDGSH iron sulfur domain 2
Down	-2.73	0.0111	CCNA1	cyclin A1
Down	-2.70	0.0084	IRAK3	interleukin-1 receptor-associated kinase 3
Down	-2.70	0.0056	MEOX2	mesenchyme homeobox 2
Down	-2.69	0.0148	VAT1L	vesicle amine transport 1-like
Down	-2.69	0.0068	STK39	serine threonine kinase 39

Supplementary Table 2

Down	-2.69	0.0079	TNFSF10	tumor necrosis factor (ligand) superfamily, member 10
Down	-2.69	0.0108	PPAT	phosphoribosyl pyrophosphate amidotransferase
Down	-2.67	0.0076	PROS1	protein S (alpha)
Down	-2.63	0.0084	NUPR1	nuclear protein, transcriptional regulator, 1
Down	-2.61	0.0058	RP11-503K16.2	
Down	-2.61	0.0101	SLC1A1	solute carrier family 1 (neuronal/epithelial high affinity glutamate transporter, system Xag), member 1
Down	-2.60	0.0096	IL10RB	interleukin 10 receptor, beta
Down	-2.56	0.0044	HPSE	heparanase
Down	-2.53	0.0068	PROSP	protein S pseudogen
Down	-2.52	0.0111	CLDN10-AS1	CLDN10 antisense RNA 1
Down	-2.50	0.0096	EFNA5	ephrin-A5
Down	-2.50	0.0064	CTSH	cathepsin H
Down	-2.49	0.0086	MLLT11	myeloid/lymphoid or mixed-lineage leukemia; translocated to, 11
Down	-2.47	0.0044	CCNA2	cyclin A2
Down	-2.46	0.0143	CXCL8	chemokine (C-X-C motif) ligand 8
Down	-2.44	0.0222	CCL15-CCL14	CCL15-CCL14 readthrough (NMD candidate)
Down	-2.43	0.0096	HERC6	HECT and RLD domain containing E3 ubiquitin protein ligase family member 6
Down	-2.41	0.0095	RASA4	RAS p21 protein activator 4
Down	-2.40	0.0068	RFTN2	raftlin family member 2
Down	-2.38	0.0107	PRKCI	protein kinase C, iota
Down	-2.35	0.0051	RASA4	RAS p21 protein activator 4
Down	-2.35	0.0059	TFPI2	tissue factor pathway inhibitor 2
Down	-2.35	0.0076	RNF168	ring finger protein 168, E3 ubiquitin protein ligase
Down	-2.34	0.0082	SLC25A30	solute carrier family 25, member 30
Down	-2.34	0.0096	CDKN3	cyclin-dependent kinase inhibitor 3
Down	-2.31	0.0252	PRKAA2	protein kinase, AMP-activated, alpha 2 catalytic subunit
Down	-2.30	0.0404	LOC100130298	
Down	-2.29	0.0059	PAICS	phosphoribosylaminoimidazole carboxylase, phosphoribosylaminoimidazole succinocarboxamide synthetase
Down	-2.26	0.0271	LOC374443	
Down	-2.26	0.0111	HIST1H3G	histone cluster 1, H3g
Down	-2.25	0.0096	SAT1	spermidine/spermine N1-acetyltransferase 1
Down	-2.24	0.0236	PROS1	protein S (alpha)
Down	-2.24	0.0076	DHCR24	24-dehydrocholesterol reductase
Down	-2.23	0.0096	HLA-DPA1	major histocompatibility complex, class II, DP alpha 1
Down	-2.23	0.0108	PROS1	protein S (alpha)
Down	-2.22	0.0203	TEX30	testis expressed 30
Down	-2.21	0.0082	SCARA3	scavenger receptor class A, member 3
Down	-2.20	0.0437	OAS2	2'-5'-oligoadenylate synthetase 2, 69/71kDa
Down	-2.20	0.0126	HLA-DPA1	major histocompatibility complex, class II, DP alpha 1
Down	-2.20	0.0126	HLA-DPA1	major histocompatibility complex, class II, DP alpha 1
Down	-2.20	0.0126	HLA-DPA1	major histocompatibility complex, class II, DP alpha 1
Down	-2.20	0.0298	TRPC6	transient receptor potential cation channel, subfamily C, member 6
Down	-2.19	0.0178	LGALS9C	lectin, galactoside-binding, soluble, 9C

Supplementary Table 2

Down	-2.19	0.0123	HLA-DPA1	major histocompatibility complex, class II, DP alpha 1
Down	-2.18	0.0189	SLC40A1	solute carrier family 40 (iron-regulated transporter), member 1
Down	-2.18	0.0118	HLA-DPA1	major histocompatibility complex, class II, DP alpha 1
Down	-2.15	0.0139	HLA-DPA1	major histocompatibility complex, class II, DP alpha 1
Down	-2.15	0.0139	HLA-DPA1	major histocompatibility complex, class II, DP alpha 1
Down	-2.14	0.0258	TESK2	testis-specific kinase 2
Down	-2.14	0.0276	RASA4B	RAS p21 protein activator 4B
Down	-2.13	0.0320	IFI44L	interferon-induced protein 44-like
Down	-2.13	0.0117	HIST1H1A	histone cluster 1, H1a
Down	-2.11	0.0360	MGST1	microsomal glutathione S-transferase 1
Down	-2.11	0.0207	TSPAN13	tetraspanin 13
Down	-2.10	0.0096	MMP16	matrix metallopeptidase 16 (membrane-inserted)
Down	-2.10	0.0092	SNCA	synuclein, alpha (non A4 component of amyloid precursor)
Down	-2.09	0.0168	C8orf88	chromosome 8 open reading frame 88
Down	-2.08	0.0096	ABCA6	ATP-binding cassette, sub-family A (ABC1), member 6
Down	-2.08	0.0096	ALOX5AP	arachidonate 5-lipoxygenase-activating protein
Down	-2.07	0.0205	ELOVL4	ELOVL fatty acid elongase 4
Down	-2.07	0.0212	KIF15	kinesin family member 15
Down	-2.07	0.0216	HIST1H3B	histone cluster 1, H3b
Down	-2.07	0.0069	PROSP	protein S pseudogen
Down	-2.06	0.0085	PYGL	phosphorylase, glycogen, liver
Down	-2.06	0.0184	SLC2A10	solute carrier family 2 (facilitated glucose transporter), member 10
Down	-2.06	0.0498	OCLN	occludin
Down	-2.06	0.0477	RASA4	RAS p21 protein activator 4
Down	-2.05	0.0076	FADS2	fatty acid desaturase 2
Down	-2.04	0.0096	MKRN2	makorin ring finger protein 2
Down	-2.03	0.0079	RASA4	RAS p21 protein activator 4
Down	-2.03	0.0088	DCAKD	dephospho-CoA kinase domain containing
Down	-2.03	0.0129	AMOT	angiomotin
Down	-2.01	0.0208	SULT1C4	sulfotransferase family, cytosolic, 1C, member 4
Down	-2.01	0.0118	SPAG5	sperm associated antigen 5
Down	-2.01	0.0096	MBOAT1	membrane bound O-acyltransferase domain containing 1
Down	-2.00	0.0086	NPM1P39	nucleophosmin 1 (nucleolar phosphoprotein B23, numatrin) pseudogene 39
Down	-2.00	0.0084	DLGAP5	discs, large (Drosophila) homolog-associated protein 5
Down	-2.00	0.0402	EHD3	EH-domain containing 3
Down	-2.00	0.0184	CYYR1	cysteine/tyrosine-rich 1
Down	-2.00	0.0126	TJP1	tight junction protein 1
Down	-1.99	0.0110	WDR13	WD repeat domain 13
Down	-1.98	0.0098	HADHB	hydroxyacyl-CoA dehydrogenase/3-ketoacyl-CoA thiolase/enoyl-CoA hydratase (trifunctional protein), beta subunit
Down	-1.98	0.0095	VPS4B	vacuolar protein sorting 4 homolog B (S. cerevisiae)
Down	-1.98	0.0095	RASA4B	RAS p21 protein activator 4B
Down	-1.97	0.0402	MTX3	metaxin 3
Down	-1.97	0.0096	CNR1	

Supplementary Table 2

Down	-1.97	0.0088	GMFG	glia maturation factor, gamma
Down	-1.97	0.0122	ZBED6	zinc finger, BED-type containing 6
Down	-1.97	0.0255	PRKD1	protein kinase D1
Down	-1.97	0.0120	RELN	reelin
Down	-1.96	0.0079	NEDD1	neural precursor cell expressed, developmentally down-regulated 1
Down	-1.96	0.0163	IMPA1	inositol(myo)-1(or 4)-monophosphatase 1
Down	-1.96	0.0165	CNR1	cannabinoid receptor 1 (brain)
Down	-1.95	0.0466	CD36	CD36 molecule (thrombospondin receptor)
Down	-1.95	0.0096	STK40	serine/threonine kinase 40
Down	-1.94	0.0111	OSBPL6	oxysterol binding protein-like 6
Down	-1.94	0.0130	GOLM1	golgi membrane protein 1
Down	-1.93	0.0175	GRAPL	GRB2-related adaptor protein-like
Down	-1.93	0.0108	THEMIS2	thymocyte selection associated family member 2
Down	-1.93	0.0288	MYO1F	myosin IF
Down	-1.93	0.0113	GGT1	gamma-glutamyltransferase 1
Down	-1.92	0.0209	NT5DC1	5'-nucleotidase domain containing 1
Down	-1.92	0.0329	PLA2G4A	phospholipase A2, group IVA (cytosolic, calcium-dependent)
Down	-1.92	0.0178	SCML1	sex comb on midleg-like 1 (Drosophila)
Down	-1.91	0.0095	PRPS2	phosphoribosyl pyrophosphate synthetase 2
Down	-1.91	0.0088	FOXM1	forkhead box M1
Down	-1.91	0.0096	SORD	sorbitol dehydrogenase
Down	-1.90	0.0096	MME	membrane metallo-endopeptidase
Down	-1.90	0.0379	PC	pyruvate carboxylase
Down	-1.90	0.0096	RNU5F-1	RNA, U5F small nuclear 1
Down	-1.90	0.0096	RASSF2	Ras association (RalGDS/AF-6) domain family member 2
Down	-1.90	0.0095	MCL1	myeloid cell leukemia 1
Down	-1.90	0.0131	CKAP2L	cytoskeleton associated protein 2-like
Down	-1.89	0.0095	HIST1H1B	histone cluster 1, H1b
Down	-1.89	0.0096	STIL	SCL/TAL1 interrupting locus
Down	-1.88	0.0294	LOC100132167	
Down	-1.88	0.0174	CHML	choroideremia-like (Rab escort protein 2)
Down	-1.88	0.0477	KCNN3	potassium channel, calcium activated intermediate/small conductance subfamily N alpha, member 3
Down	-1.88	0.0113	CDC20	cell division cycle 20
Down	-1.88	0.0189	ULK4P1	ULK4 pseudogene 1
Down	-1.88	0.0122	AURKB	aurora kinase B
Down	-1.87	0.0096	RASA4	RAS p21 protein activator 4
Down	-1.87	0.0189	NCOA7	nuclear receptor coactivator 7
Down	-1.87	0.0165	PBK	PDZ binding kinase
Down	-1.87	0.0128	MPZL3	myelin protein zero-like 3
Down	-1.87	0.0096	RNF144B	ring finger protein 144B
Down	-1.86	0.0367	GLT8D2	glycosyltransferase 8 domain containing 2
Down	-1.86	0.0320	OAS3	2'-5'-oligoadenylate synthetase 3, 100kDa
Down	-1.86	0.0150	SLFN5	schlafen family member 5
Down	-1.86	0.0096	RAVER2	ribonucleoprotein, PTB-binding 2
Down	-1.85	0.0096	IL31RA	interleukin 31 receptor A

Supplementary Table 2

Down	-1.85	0.0096	TST	thiosulfate sulfurtransferase (rhodanese)
Down	-1.85	0.0096	TAB3	TGF-beta activated kinase 1/MAP3K7 binding protein 3
Down	-1.85	0.0118	GPR4	G protein-coupled receptor 4
Down	-1.85	0.0212	IL1R1	interleukin 1 receptor, type I
Down	-1.85	0.0096	ST6GALNAC3	ST6 (alpha-N-acetyl-neuraminyl-2,3-beta-galactosyl-1,3)-N-acetylgalactosaminide alpha-2,6-sialyltransferase 3
Down	-1.85	0.0152	KIF14	kinesin family member 14
Down	-1.84	0.0096	AOX1	aldehyde oxidase 1
Down	-1.84	0.0113	TRPV2	transient receptor potential cation channel, subfamily V, member 2
Down	-1.84	0.0184	SPC24	SPC24, NDC80 kinetochore complex component
Down	-1.84	0.0096	ABCG1	ATP-binding cassette, sub-family G (WHITE), member 1
Down	-1.84	0.0096	SLFN11	schlafen family member 11
Down	-1.83	0.0147	HLA-DPB1	major histocompatibility complex, class II, DP beta 1
Down	-1.83	0.0202	PGAP1	post-GPI attachment to proteins 1
Down	-1.83	0.0096	LAPTM5	lysosomal protein transmembrane 5
Down	-1.83	0.0207	CD163L1	CD163 molecule-like 1
Down	-1.83	0.0348	RASSF9	Ras association (RalGDS/AF-6) domain family (N-terminal) member 9
Down	-1.83	0.0252	ISOC1	isochorismatase domain containing 1
Down	-1.83	0.0239	TMEM71	transmembrane protein 71
Down	-1.83	0.0136	NPM1P21	nucleophosmin 1 (nucleolar phosphoprotein B23, numatrin) pseudogene 21
Down	-1.82	0.0096	KIF20A	kinesin family member 20A
Down	-1.82	0.0096	TMEM47	transmembrane protein 47
Down	-1.82	0.0122	MAOA	monoamine oxidase A
Down	-1.82	0.0203	ULK4P3	ULK4 pseudogene 3
Down	-1.82	0.0155	RPIA	ribose 5-phosphate isomerase A
Down	-1.82	0.0351	RNF7	ring finger protein 7
Down	-1.81	0.0096	SCD	stearoyl-CoA desaturase (delta-9-desaturase)
Down	-1.81	0.0129	TOM1L1	target of myb1 like 1 membrane trafficking protein
Down	-1.81	0.0135	TNFRSF1B	tumor necrosis factor receptor superfamily, member 1B
Down	-1.81	0.0096	ETS1	v-ets avian erythroblastosis virus E26 oncogene homolog 1
Down	-1.81	0.0307	TRIL	TLR4 interactor with leucine-rich repeats
Down	-1.81	0.0147	GABBR2	gamma-aminobutyric acid (GABA) B receptor, 2
Down	-1.81	0.0222	RCBTB2	regulator of chromosome condensation (RCC1) and BTB (POZ) domain containing protein 2
Down	-1.80	0.0107	ERMP1	endoplasmic reticulum metallopeptidase 1
Down	-1.80	0.0096	PTPRK	protein tyrosine phosphatase, receptor type, K
Down	-1.80	0.0382	BARD1	BRCA1 associated RING domain 1
Down	-1.80	0.0122	TP53I3	tumor protein p53 inducible protein 3
Down	-1.80	0.0096	PTPN9	protein tyrosine phosphatase, non-receptor type 9
Down	-1.79	0.0313	APLF	aprataxin and PNKP like factor
Down	-1.79	0.0110	EMP2	epithelial membrane protein 2
Down	-1.79	0.0154	APOD	apolipoprotein D
Down	-1.78	0.0096	HLA-DPB1	major histocompatibility complex, class II, DP beta 1
Down	-1.78	0.0096	HLA-DPB1	major histocompatibility complex, class II, DP beta 1
Down	-1.78	0.0169	HIST1H1C	histone cluster 1, H1c

Supplementary Table 2

Down	-1.78	0.0152	RALGPS2	Ral GEF with PH domain and SH3 binding motif 2
Down	-1.78	0.0172	CENPI	centromere protein I
Down	-1.78	0.0096	LTBP1	latent transforming growth factor beta binding protein 1
Down	-1.77	0.0112	SEC23A	Sec23 homolog A (S. cerevisiae)
Down	-1.77	0.0293	MCM2	minichromosome maintenance complex component 2
Down	-1.77	0.0429	ZFHX4-AS1	ZFHX4 antisense RNA 1
Down	-1.77	0.0189	HELLS	helicase, lymphoid-specific
Down	-1.77	0.0096	PRKACB	protein kinase, cAMP-dependent, catalytic, beta
Down	-1.77	0.0096	NLK	nemo-like kinase
Down	-1.77	0.0118	HSD11B1	hydroxysteroid (11-beta) dehydrogenase 1
Down	-1.77	0.0170	LAYN	layilin
Down	-1.77	0.0362	C10orf10	chromosome 10 open reading frame 10
Down	-1.77	0.0177	TMEM54	transmembrane protein 54
Down	-1.77	0.0145	MANSC4	MANSC domain containing 4
Down	-1.76	0.0133	CCNB1	cyclin B1
Down	-1.76	0.0321	HSD17B14	hydroxysteroid (17-beta) dehydrogenase 14
Down	-1.76	0.0096	NOX4	NADPH oxidase 4
Down	-1.76	0.0310	KDELR3	KDEL (Lys-Asp-Glu-Leu) endoplasmic reticulum protein retention receptor 3
Down	-1.76	0.0192	YES1	YES proto-oncogene 1, Src family tyrosine kinase
Down	-1.76	0.0096	TOP2A	topoisomerase (DNA) II alpha 170kDa
Down	-1.75	0.0145	STX11	syntaxin 11
Down	-1.75	0.0310	HIST1H2AB	histone cluster 1, H2ab
Down	-1.75	0.0122	ME1	malic enzyme 1, NADP(+)-dependent, cytosolic
Down	-1.75	0.0308	SLC7A2	solute carrier family 7 (cationic amino acid transporter, y+ system), member 2
Down	-1.75	0.0399	SLC7A11	solute carrier family 7 (anionic amino acid transporter light chain, xc- system), member 11
Down	-1.75	0.0165	AFF2	AF4/FMR2 family, member 2
Down	-1.75	0.0118	PLA2G16	phospholipase A2, group XVI
Down	-1.75	0.0331	KIF20B	kinesin family member 20B
Down	-1.75	0.0364	HIST1H2BI	histone cluster 1, H2bi
Down	-1.74	0.0096	PITPNC1	phosphatidylinositol transfer protein, cytoplasmic 1
Down	-1.74	0.0118	HIST1H2AI	histone cluster 1, H2ai
Down	-1.74	0.0118	PDE8A	phosphodiesterase 8A
Down	-1.74	0.0288	C12orf66	chromosome 12 open reading frame 66
Down	-1.74	0.0123	RAB3GAP1	RAB3 GTPase activating protein subunit 1 (catalytic)
Down	-1.74	0.0227	FAM83D	family with sequence similarity 83, member D
Down	-1.74	0.0256	ZNF451	zinc finger protein 451
Down	-1.74	0.0123	BVES	blood vessel epicardial substance
Down	-1.73	0.0096	TMEM170A	transmembrane protein 170A
Down	-1.73	0.0154	KIF18A	kinesin family member 18A
Down	-1.73	0.0409	TTLL7	tubulin tyrosine ligase-like family member 7
Down	-1.73	0.0212	DEPDC1	DEP domain containing 1
Down	-1.73	0.0222	SPIN4	spindlin family, member 4
Down	-1.73	0.0096	ANLN	anillin actin binding protein
Down	-1.73	0.0148	PSRC1	proline/serine-rich coiled-coil 1
Down	-1.73	0.0240	PLLP	plasmolipin

Supplementary Table 2

Down	-1.73	0.0184	SORD	sorbitol dehydrogenase
Down	-1.73	0.0150	BMX	BMX non-receptor tyrosine kinase
Down	-1.73	0.0133	GJD3	gap junction protein, delta 3, 31.9kDa
Down	-1.73	0.0203	HLA-DPB1	major histocompatibility complex, class II, DP beta 1
Down	-1.73	0.0130	MST4	serine/threonine protein kinase 26
Down	-1.73	0.0165	LINC01235	long intergenic non-protein coding RNA 1235
Down	-1.72	0.0207	BCRP3	breakpoint cluster region pseudogene 3
Down	-1.72	0.0187	CLU	clusterin
Down	-1.72	0.0172	ZBED6	zinc finger, BED-type containing 6
Down	-1.72	0.0288	PABPC4L	poly(A) binding protein, cytoplasmic 4-like
Down	-1.72	0.0108	FZD4	frizzled class receptor 4
Down	-1.72	0.0189	TNFAIP3	tumor necrosis factor, alpha-induced protein 3
Down	-1.72	0.0498	PLK4	polo-like kinase 4
Down	-1.72	0.0130	STK4	serine/threonine kinase 4
Down	-1.72	0.0214	HLA-DPB1	major histocompatibility complex, class II, DP beta 1
Down	-1.72	0.0214	HLA-DPB1	major histocompatibility complex, class II, DP beta 1
Down	-1.72	0.0214	HLA-DPB1	major histocompatibility complex, class II, DP beta 1
Down	-1.72	0.0145	B3GALNT2	beta-1,3-N-acetylgalactosaminyltransferase 2
Down	-1.72	0.0108	SCARB2	scavenger receptor class B, member 2
Down	-1.72	0.0214	SCG5	secretogranin V
Down	-1.71	0.0118	CDC7	cell division cycle 7
Down	-1.71	0.0096	ACTR2	ARP2 actin-related protein 2 homolog (yeast)
Down	-1.71	0.0219	DOCK11	dedicator of cytokinesis 11
Down	-1.71	0.0489	CDK1	cyclin-dependent kinase 1
Down	-1.71	0.0127	CDCP1	CUB domain containing protein 1
Down	-1.71	0.0147	STOM	stomatin
Down	-1.71	0.0428	SLC22A23	solute carrier family 22, member 23
Down	-1.70	0.0310	RP11-445N18.5	
Down	-1.70	0.0439	RNF144A-AS1	RNF144A antisense RNA 1
Down	-1.70	0.0195	HIST1H1D	histone cluster 1, H1d
Down	-1.70	0.0214	C1orf21	chromosome 1 open reading frame 21
Down	-1.70	0.0173	PNPO	pyridoxamine 5'-phosphate oxidase
Down	-1.69	0.0243	CDC6	cell division cycle 6
Down	-1.69	0.0157	KIAA1524	KIAA1524
Down	-1.69	0.0096	OCIAD1	OCIA domain containing 1
Down	-1.69	0.0184	ARNTL2	aryl hydrocarbon receptor nuclear translocator-like 2
Down	-1.69	0.0115	CYCS	cytochrome c, somatic
Down	-1.69	0.0256	EIF5A2	eukaryotic translation initiation factor 5A2
Down	-1.69	0.0111	BIRC5	baculoviral IAP repeat containing 5
Down	-1.69	0.0208	COBLL1	cordon-bleu WH2 repeat protein-like 1
Down	-1.69	0.0170	SLC1A4	solute carrier family 1 (glutamate/neutral amino acid transporter), member 4
Down	-1.69	0.0128	HSD17B4	hydroxysteroid (17-beta) dehydrogenase 4
Down	-1.69	0.0118	EMC10	ER membrane protein complex subunit 10
Down	-1.69	0.0113	DTL	denticleless E3 ubiquitin protein ligase homolog (Drosophila)
Down	-1.69	0.0230	MIR4263	microRNA 4263

Supplementary Table 2

Down	-1.68	0.0101	WASL	Wiskott-Aldrich syndrome-like
Down	-1.68	0.0232	USP13	ubiquitin specific peptidase 13 (isopeptidase T-3)
Down	-1.68	0.0241	ADD3	adducin 3 (gamma)
Down	-1.68	0.0143	MYLK	myosin light chain kinase
Down	-1.68	0.0288	FAM46A	family with sequence similarity 46, member A
Down	-1.68	0.0118	FADS1	fatty acid desaturase 1
Down	-1.68	0.0152	TFRC	transferrin receptor
Down	-1.67	0.0237	EPN2	epsin 2
Down	-1.67	0.0411	NEIL3	nei-like DNA glycosylase 3
Down	-1.67	0.0434	IGFBP1	insulin-like growth factor binding protein 1
Down	-1.67	0.0152	EPS8	epidermal growth factor receptor pathway substrate 8
Down	-1.67	0.0123	PRKAR2B	protein kinase, cAMP-dependent, regulatory, type II, beta
Down	-1.67	0.0331	ARG2	arginase 2
Down	-1.67	0.0118	NCAPG	non-SMC condensin I complex, subunit G
Down	-1.67	0.0447	NCR3LG1	natural killer cell cytotoxicity receptor 3 ligand 1
Down	-1.67	0.0150	NRIP3	nuclear receptor interacting protein 3
Down	-1.66	0.0113	NQO1	NAD(P)H dehydrogenase, quinone 1
Down	-1.66	0.0201	TBC1D30	TBC1 domain family, member 30
Down	-1.66	0.0308	SUDS3	suppressor of defective silencing 3 homolog (S. cerevisiae)
Down	-1.66	0.0243	RASA4	RAS p21 protein activator 4
Down	-1.66	0.0107	RNPS1	RNA binding protein S1, serine-rich domain
Down	-1.66	0.0256	NOX4	NADPH oxidase 4
Down	-1.66	0.0353	ACTR1B	ARP1 actin-related protein 1 homolog B, centractin beta (yeast)
Down	-1.66	0.0133	NIPAL2	NIPA-like domain containing 2
Down	-1.65	0.0214	ARL4A	ADP-ribosylation factor-like 4A
Down	-1.65	0.0349	IL1A	interleukin 1, alpha
Down	-1.65	0.0327	MRC2	mannose receptor, C type 2
Down	-1.65	0.0129	NCAPH	non-SMC condensin I complex, subunit H
Down	-1.65	0.0277	SQRDL	sulfide quinone reductase-like (yeast)
Down	-1.65	0.0230	KLHL8	kelch-like family member 8
Down	-1.65	0.0246	ARHGEF6	Rac/Cdc42 guanine nucleotide exchange factor (GEF) 6
Down	-1.65	0.0230	SLC39A8	solute carrier family 39 (zinc transporter), member 8
Down	-1.65	0.0222	CNTNAP1	contactin associated protein 1
Down	-1.65	0.0173	NUF2	NUF2, NDC80 kinetochore complex component
Down	-1.65	0.0494	RP3-475N16.1	
Down	-1.65	0.0112	PRC1	protein regulator of cytokinesis 1
Down	-1.65	0.0207	MCM10	minichromosome maintenance complex component 10
Down	-1.64	0.0314	ROPN1L	rhophilin associated tail protein 1-like
Down	-1.64	0.0123	SGCB	sarcoglycan, beta (43kDa dystrophin-associated glycoprotein)
Down	-1.64	0.0222	TRERF1	transcriptional regulating factor 1
Down	-1.64	0.0459	CDKN2C	cyclin-dependent kinase inhibitor 2C (p18, inhibits CDK4)
Down	-1.64	0.0236	SESN3	sestrin 3
Down	-1.64	0.0150	ZDHHC20	zinc finger, DHHC-type containing 20
Down	-1.64	0.0226	DCTN2	dynactin 2 (p50)
Down	-1.64	0.0364	SCN5A	sodium channel, voltage gated, type V alpha subunit

Supplementary Table 2

Down	-1.64	0.0118	PLK1	polo-like kinase 1
Down	-1.64	0.0345	DNAJC6	DnaJ (Hsp40) homolog, subfamily C, member 6
Down	-1.64	0.0278	TMEM126A	transmembrane protein 126A
Down	-1.64	0.0117	DYNLL2	dynein, light chain, LC8-type 2
Down	-1.63	0.0214	H6PD	hexose-6-phosphate dehydrogenase (glucose 1-dehydrogenase)
Down	-1.63	0.0111	ERC1	ELKS/RAB6-interacting/CAST family member 1
Down	-1.63	0.0258	F2RL2	coagulation factor II (thrombin) receptor-like 2
Down	-1.63	0.0154	UHRF1	ubiquitin-like with PHD and ring finger domains 1
Down	-1.63	0.0172	RAET1E	retinoic acid early transcript 1E
Down	-1.63	0.0230	NOX4	NADPH oxidase 4
Down	-1.63	0.0169	FYTTD1	forty-two-three domain containing 1
Down	-1.63	0.0130	GAS2L3	growth arrest-specific 2 like 3
Down	-1.62	0.0136	HIST1H4D	histone cluster 1, H4d
Down	-1.62	0.0184	KLHL4	kelch-like family member 4
Down	-1.62	0.0214	RP11-555H23.1	
Down	-1.62	0.0426	LINC01268	long intergenic non-protein coding RNA 1268
Down	-1.62	0.0380	FBN2	fibrillin 2
Down	-1.62	0.0145	SPHK1	sphingosine kinase 1
Down	-1.62	0.0212	NPM1P21	nucleophosmin 1 (nucleolar phosphoprotein B23, numatrin) pseudogene 21
Down	-1.62	0.0304	MFSD6	major facilitator superfamily domain containing 6
Down	-1.62	0.0118	TMEM245	transmembrane protein 245
Down	-1.62	0.0154	RPS6KA3	ribosomal protein S6 kinase, 90kDa, polypeptide 3
Down	-1.62	0.0350	LINC00639	long intergenic non-protein coding RNA 639
Down	-1.62	0.0476	GLCCI1	glucocorticoid induced 1
Down	-1.62	0.0428	FERMT3	fermitin family member 3
Down	-1.62	0.0434	MIR146A	microRNA 146a
Down	-1.62	0.0256	DPYSL3	dihydropyrimidinase-like 3
Down	-1.61	0.0150	PHF19	PHD finger protein 19
Down	-1.61	0.0117	RNPS1	RNA binding protein S1, serine-rich domain
Down	-1.61	0.0439	GPR1	G protein-coupled receptor 1
Down	-1.61	0.0159	GTSE1	G-2 and S-phase expressed 1
Down	-1.61	0.0154	PTPLA	3-hydroxyacyl-CoA dehydratase 1
Down	-1.61	0.0295	TROAP	trophinin associated protein
Down	-1.60	0.0280	CYP2U1	cytochrome P450, family 2, subfamily U, polypeptide 1
Down	-1.60	0.0170	CTNNAL1	catenin (cadherin-associated protein), alpha-like 1
Down	-1.60	0.0126	RRM2	ribonucleotide reductase M2
Down	-1.60	0.0274	CYP1A1	cytochrome P450, family 1, subfamily A, polypeptide 1
Down	-1.60	0.0327	ITGA8	integrin, alpha 8
Down	-1.60	0.0192	TYMS	thymidylate synthetase
Down	-1.60	0.0442	UBE2C	ubiquitin-conjugating enzyme E2C
Down	-1.60	0.0169	NETO2	neuropilin (NRP) and tolloid (TLL)-like 2
Down	-1.60	0.0295	PCOLCE	procollagen C-endopeptidase enhancer
Down	-1.60	0.0351	MYH10	myosin, heavy chain 10, non-muscle
Down	-1.60	0.0150	HIST2H3A	histone cluster 2, H3a
Down	-1.60	0.0150	HIST2H3A	histone cluster 2, H3a

Supplementary Table 2

Down	-1.60	0.0177	TPX2	TPX2, microtubule-associated
Down	-1.60	0.0235	ATL1	atlastin GTPase 1
Down	-1.60	0.0314	FAM162B	family with sequence similarity 162, member B
Down	-1.59	0.0187	NEK9	NIMA-related kinase 9
Down	-1.59	0.0240	NPM1P39	nucleophosmin 1 (nucleolar phosphoprotein B23, numatrin) pseudogene 39
Down	-1.59	0.0176	ITPR2	inositol 1,4,5-trisphosphate receptor, type 2
Down	-1.59	0.0222	CDCA7L	cell division cycle associated 7-like
Down	-1.59	0.0434	GPR3	G protein-coupled receptor 3
Down	-1.59	0.0149	HNRNPD	heterogeneous nuclear ribonucleoprotein D (AU-rich element RNA binding protein 1, 37kDa)
Down	-1.59	0.0177	ADK	adenosine kinase
Down	-1.58	0.0480	RASA4	RAS p21 protein activator 4
Down	-1.58	0.0129	GREM1	gremlin 1, DAN family BMP antagonist
Down	-1.58	0.0338	TMOD2	tropomodulin 2 (neuronal)
Down	-1.58	0.0148	CDKAL1	CDK5 regulatory subunit associated protein 1-like 1
Down	-1.58	0.0301	EPB41L5	erythrocyte membrane protein band 4.1 like 5
Down	-1.58	0.0465	RASA4CP	RAS p21 protein activator 4C, pseudogene
Down	-1.58	0.0197	FAM171B	family with sequence similarity 171, member B
Down	-1.58	0.0184	FAM98A	family with sequence similarity 98, member A
Down	-1.58	0.0425	P4HA3	prolyl 4-hydroxylase, alpha polypeptide III
Down	-1.58	0.0233	TPMT	thiopurine S-methyltransferase
Down	-1.58	0.0256	SIRPA	signal-regulatory protein alpha
Down	-1.58	0.0236	C20orf194	chromosome 20 open reading frame 194
Down	-1.58	0.0149	TACC1	transforming, acidic coiled-coil containing protein 1
Down	-1.58	0.0152	RNPS1	RNA binding protein S1, serine-rich domain
Down	-1.58	0.0285	METTL7A	methyltransferase like 7A
Down	-1.58	0.0178	TMEM97	transmembrane protein 97
Down	-1.58	0.0149	RP11-4C20.4	
Down	-1.57	0.0164	PSIP1	PC4 and SFRS1 interacting protein 1
Down	-1.57	0.0310	CDC25C	cell division cycle 25C
Down	-1.57	0.0154	FAM213A	family with sequence similarity 213, member A
Down	-1.57	0.0409	CENPW	centromere protein W
Down	-1.57	0.0399	CDCA2	cell division cycle associated 2
Down	-1.57	0.0169	SKA3	spindle and kinetochore associated complex subunit 3
Down	-1.57	0.0409	UBE2S	ubiquitin-conjugating enzyme E2S
Down	-1.57	0.0190	ALCAM	activated leukocyte cell adhesion molecule
Down	-1.57	0.0135	LPCAT3	lysophosphatidylcholine acyltransferase 3
Down	-1.57	0.0152	KIF11	kinesin family member 11
Down	-1.57	0.0274	CPA3	carboxypeptidase A3 (mast cell)
Down	-1.57	0.0190	SEC11A	SEC11 homolog A (S. cerevisiae)
Down	-1.57	0.0295	CTDSP2	CTD (carboxy-terminal domain, RNA polymerase II, polypeptide A) small phosphatase 2
Down	-1.57	0.0320	HMGCS1	3-hydroxy-3-methylglutaryl-CoA synthase 1 (soluble)
Down	-1.57	0.0184	HIST1H2BM	histone cluster 1, H2bm
Down	-1.56	0.0353	CTSO	cathepsin O
Down	-1.56	0.0423	ANKRD55	ankyrin repeat domain 55
Down	-1.56	0.0444	ARHGAP11A	Rho GTPase activating protein 11A

Supplementary Table 2

Down	-1.56	0.0245	INTS7	integrator complex subunit 7
Down	-1.56	0.0189	CDCA8	cell division cycle associated 8
Down	-1.55	0.0268	HIST1H3F	histone cluster 1, H3f
Down	-1.55	0.0307	FGD4	FYVE, RhoGEF and PH domain containing 4
Down	-1.55	0.0399	STAC	SH3 and cysteine rich domain
Down	-1.55	0.0427	NUDT15	nudix (nucleoside diphosphate linked moiety X)-type motif 15
Down	-1.55	0.0152	MYL9	myosin, light chain 9, regulatory
Down	-1.55	0.0301	GRAP	GRB2-related adaptor protein
Down	-1.55	0.0400	TPI1P2	triosephosphate isomerase 1 pseudogene 2
Down	-1.55	0.0307	TUSC3	tumor suppressor candidate 3
Down	-1.55	0.0270	EPHA5	EPH receptor A5
Down	-1.55	0.0337	NOX4	NADPH oxidase 4
Down	-1.55	0.0314	NDRG4	NDRG family member 4
Down	-1.55	0.0175	AHR	aryl hydrocarbon receptor
Down	-1.55	0.0361	RNF125	ring finger protein 125, E3 ubiquitin protein ligase
Down	-1.55	0.0292	TMEM9B	TMEM9 domain family, member B
Down	-1.54	0.0399	MAD2L1	MAD2 mitotic arrest deficient-like 1 (yeast)
Down	-1.54	0.0409	SORT1	sortilin 1
Down	-1.54	0.0314	BRIP1	BRCA1 interacting protein C-terminal helicase 1
Down	-1.54	0.0400	MYO5C	myosin VC
Down	-1.54	0.0227	NCAPG2	non-SMC condensin II complex, subunit G2
Down	-1.54	0.0149	NDFIP1	Nedd4 family interacting protein 1
Down	-1.54	0.0227	HOXD1	homeobox D1
Down	-1.54	0.0198	IL32	interleukin 32
Down	-1.54	0.0253	IQGAP3	IQ motif containing GTPase activating protein 3
Down	-1.54	0.0436	C1orf110	chromosome 1 open reading frame 110
Down	-1.54	0.0297	FBXW8	F-box and WD repeat domain containing 8
Down	-1.54	0.0425	KIF2C	kinesin family member 2C
Down	-1.54	0.0307	THSD4	thrombospondin, type I, domain containing 4
Down	-1.54	0.0255	EYA1	EYA transcriptional coactivator and phosphatase 1
Down	-1.54	0.0366	SGOL1	shugoshin-like 1 (S. pombe)
Down	-1.54	0.0352	HJURP	Holliday junction recognition protein
Down	-1.53	0.0362	LRP8	low density lipoprotein receptor-related protein 8, apolipoprotein e receptor
Down	-1.53	0.0149	RBMS2	RNA binding motif, single stranded interacting protein 2
Down	-1.53	0.0434	TTLL11-IT1	TTLL11 intronic transcript 1
Down	-1.53	0.0363	TMPO-AS1	TMPO antisense RNA 1
Down	-1.53	0.0223	CBX6	chromobox homolog 6
Down	-1.53	0.0191	TTK	TTK protein kinase
Down	-1.53	0.0366	SUCLG2	succinate-CoA ligase, GDP-forming, beta subunit
Down	-1.53	0.0277	CLDN10	claudin 10
Down	-1.53	0.0391	STARD3NL	STARD3 N-terminal like
Down	-1.53	0.0436	PHKA1	phosphorylase kinase, alpha 1 (muscle)
Down	-1.53	0.0170	NQO2	NAD(P)H dehydrogenase, quinone 2
Down	-1.53	0.0379	CHAF1B	chromatin assembly factor 1, subunit B (p60)
Down	-1.53	0.0458	GPR162	G protein-coupled receptor 162

Supplementary Table 2

Down	-1.53	0.0198	ARSB	arylsulfatase B
Down	-1.52	0.0361	KIFC1	kinesin family member C1
Down	-1.52	0.0241	KDM1B	lysine (K)-specific demethylase 1B
Down	-1.52	0.0252	ACAT2	acetyl-CoA acetyltransferase 2
Down	-1.52	0.0295	KCNAB1	potassium channel, voltage gated subfamily A regulatory beta subunit 1
Down	-1.52	0.0258	REL	v-rel avian reticuloendotheliosis viral oncogene homolog
Down	-1.52	0.0418	SEL1L	sel-1 suppressor of lin-12-like (C. elegans)
Down	-1.52	0.0177	TMEM241	transmembrane protein 241
Down	-1.52	0.0446	MT1F	metallothionein 1F
Down	-1.52	0.0295	CNTRL	centriolin
Down	-1.52	0.0313	CKS1B	CDC28 protein kinase regulatory subunit 1B
Down	-1.51	0.0407	KIFC1	kinesin family member C1
Down	-1.51	0.0407	KIFC1	kinesin family member C1
Down	-1.51	0.0298	LY75-CD302	LY75-CD302 readthrough
Down	-1.51	0.0176	HIST2H2AB	histone cluster 2, H2ab
Down	-1.51	0.0251	CNTLN	centlein, centrosomal protein
Down	-1.51	0.0348	TRIP13	thyroid hormone receptor interactor 13
Down	-1.51	0.0251	UBE2T	ubiquitin-conjugating enzyme E2T
Down	-1.51	0.0307	FAM111A	family with sequence similarity 111, member A
Down	-1.51	0.0309	SENP2	SUMO1/sentrin/SMT3 specific peptidase 2
Down	-1.51	0.0255	GIMAP4	GTPase, IMAP family member 4
Down	-1.51	0.0208	HIST1H2AE	histone cluster 1, H2ae
Down	-1.51	0.0277	CTD-2291D10.4	
Down	-1.50	0.0233	HEBP1	heme binding protein 1
Down	-1.50	0.0227	ETV4	ets variant 4
Down	-1.50	0.0313	TERC	telomerase RNA component
Down	-1.50	0.0288	KLHL5	kelch-like family member 5
Down	-1.50	0.0244	ANXA3	annexin A3
Down	-1.50	0.0255	MMAB	methylmalonic aciduria (cobalamin deficiency) cblB type

Reg : regulation, FC : Fold-change. Some genes appear more than once. Genes are sorted by fold-change.

Supplementary table 3: List of primers used for PCR and Quantitative PCR

Primer	Forward	Reward
**MOUSE**		
**Target genes**		
Ace (ACE)	TGTCGCCTCCGCTCTTGAT	CAACTCCTTGGCCTTCTTGC
Agtr1a (AT1a)	ATTCAACGCTCCCCATAGGA	TGAATTTCATAAGCCTTCTTTAGAGCT
Agt (AGT)	GTGCGGAGGCAAATCTGAAC	TGCTCGTAGATGGCGAACAG
Atp1a1 (Na/K α1)	CGTGGATAACCTCTGCTTCGT	ATGGCTTTCGCTGTGATTGG
Atp1b1 (Na/K β1)	GTGATGGGTTGTGTTGTGCTC	TCATAAGGACGCAGCACCAG
Ccl2 (MCP1)	AGCACCAGCCAACTCTCACT	TCTGGACCCATTCCTTTCTTG
Ccl5 (Rantes)	GTGCCCACGTCAAGGAGTAT	TTCTCTGGGTTGGCACACAC
Ddc (DDC)	TCCCCAGAGTTCACACAAGC	CGAAGGGCAGAAGCTCTCAT
Drd1 (DRD1)	ATGGGGGTATTCGTGTGCTG	TCAATGCAGAATGGCTGGGT
Et1 (ET1)	GCCACAGACCAGGCAGTTAG	CGAAAAGATGCCTTGATGCTATT
Grk4 (GRK4)	TGAAGTTTCCCCCAGTCAGC	TCGTACTCTGCCACTGCATC
Havcr1 (KIM1)	GCTGCTACTGCTCCTTGTGA	GGAAGGCAACCACGCTTAGA
Nphs1 (Nephrin)	CTGCATAGCCAGAGGTGGAA	CAGGATGGTAACGGCACTGG
Nphs2 (Podocin)	CACAAAGACAGGCCAAAGTGC	TTGATGCTCCCTTGTGCTCTG
Nppa (ANP)	GTAGGATTGACAGGATTGG	TGATAGATGAAGGCAGGA
Pai1 (PAI1)	AGCTTTGTGAAGGAGGACCG	CTGATGGGCTGTGTGGGATT
Ren (Renin)	GTTTCCTCAGCCAGGACTCG	AAGCCCATGCCTAGAACACC
Slc4a4 (Nbce1)	CTTGACCGGGAGGCATCTTC	ACATCCTACTTGCACTGGAGAC
Slc5a2 (Sglt2)	TGTATCTGACCGCTGGTGTG	ATGGTGATGCCCAAGAGAGC
Scnn1a (ENaC-α)	TACGCGACAACAATCCCCAA	TCCACCCCGGATGAGTATGT
Scnn1b (ENaC-β)	CACACTGGAGCAGCTTCCTAA	CCCCTCACAGATGATGCGTT
Scnn1g (ENaC-γ)	CTGAGCGAACCTTACAGCCA	GTTGGGGTGTTGCTGGTAGT
Slc9a3 (NHE3)	AGCTATGTGGCTGAGGGAGA	CCAGGCATACAGCACTGACA
Slc9a1 (NHE1)	AACGGCTGCGGTCCTATAAC	GGTTCATAGGCCAGTGGGTC
Slc34a1 (Napi-2a)	CTATCCAACCCAGTGGCAGG	TGTTGGTGACAGAGGTTCCG
**Reference genes**		
Gapdh (GAPDH)	AATGGTGAAGGTCGGTGTG	GAAGATGGTGATGGGCTTCC
Hprt (HPRT)	CTCAACTTTAACTGGAAAGAATGTC	TCCTTTTCACCAGCAAGCT
RS16	CAAGTTACTGGAGCCTGTT	GAGATGGACTGTCGGATG
Ubc (UBC)	AGCCCAGTGTTACCACCAAG	ACCCAAGAACAAGCACAAGG
**HUMAN**		
**Target genes**		
BMP4 (BMP4)	CCACAGCACTGGTCTTGAGTA	AGCAGAGTTTTCACTGGTCCC
ICAM1 (ICAM1)	GGTAGCAGCCGCAGTCATAA	GATAGGTTCAGGGAGGCGTG
GATA5 (GATA5)	TACCACAAGATGAATGGCGT	TTCCGTGTCTGGATGCTTTC
IL6 (IL6)	CCACCGGGAACGAAAGAGAA	GAGAAGGCAACTGGACCGAA
NOS3 (NOS3)	GTCCAGTAACACAGACAGTGC	CTCCCACACAGAAGGTCTCA
PRKAA2 (AMP-Cα2)	TGACCCACTGAAACGAGCA	GGAGGACTAGAGGCGAGGTA
PRKACB (PKA-Cβ)	TCCCCCATTCTTTGCAGACC	CGTGGCAAACCACTTGTGAG
PRKAR2B (PKA-R2β)	AGGCAGGATGAGCATCGAGA	CACGCCTTGTGAATCGGTTT
**Reference genes**		
GAPDH (GAPDH)	TCAAGGCTGAGAACGGGAAG	TCGCCCCACTTGATTTTGGA
HPRT (HPRT)	GACCCCACGAAGTGTTGGATA	AGCAGATGGCCACAGAACTA
UBC (UBC)	TTTCCAGAGAGCGGAACAGG	AGAACTGCGACCCAAATCCC

Supplementary Table 4. Clinical characteristics of patients taking and not taking medication for hypertension in the combined OHGS A2 and B2 GWAS

	**Taking HTN (n=2688)**	**n**	**Not taking HTN (n=3147)**	**n**	***p***
Age, (years)	65.5 ± 12.01	2321	68.8 ± 10.5	2692	<0.0001
Male sex, n (%)	1527 (56.8)	2688	2105 (66.9)	3147	<0.0001
BMI, kg/m²	28.3 ± 5.08	2686	26.7 ± 4.52	3147	<0.0001
Smoking, n (%)	1696 (63.2)	2685	1870 (59.5)	3145	3.90E-03
CAD, n (%)	1192 (44.4)	2688	1553 (49.4)	3147	1.36E-04
Systolic BP	139 ± 18.9	2615	132 ± 17.9	3070	<0.0001
Diastolic BP	78 ± 11.2	2615	76 ± 10.1	3069	<0.0001
Pulse pressure	60.8 ± 16.5	2615	56.4 ± 15.1	3069	<0.0001

Continuous variables presented as mean ± SD. Abbreviations: BMI, body mass index; HTN, hypertensive medication; BP, blood pressure. Smoking variable is ever/never smoker. Student's t-test performed on continuous variables and a chi-square test was performed on categorical variables.

Supplementary Table 5. Clinical characteristics of ADVANCE patients taking and not taking medication for hypertension in the GWAS

	**Taking HTN (n=1093)**	**n**	**Not taking HTN (n=1208)**	**n**	***p***
Age, (years)	67.2 ± 7.09	1093	67.22 ± 6.47	1208	<0.96
Male sex, n (%)	718 (66)	1093	828 (68.5)	1208	<0.15
BMI, kg/m²	30.6 ± 5.01	1093	29.17± 4.99	1208	<0.0001
Smoking, n (%)	159 (15)	1093	248 (20.5)	1208	0.0002
CAD, N (%)	906 (83)	1093	326 (27)	1208	<0.0001
Systolic BP	150.24 ± 21.73	1093	142.7 ± 19.23	1208	<0.0001
Diastolic BP	82.75 ± 11.27	1093	80.14± 9.73	1208	<0.0001
Pulse pressure	67.49 ± 17.37	1093	62.57 ± 15.28	1195	<0.0001

Continuous variables presented as mean ± SD. Abbreviations: BMI, body mass index; HTN, hypertensive medication.
